# Supplementary material for: Atrial-like Engineered Heart Tissue: An In Vitro Model of the Human Atrium
Source: Stem Cell Reports. 2018 Nov 8;11(6):1378–90. doi: 10.1016/j.stemcr.2018.10.008 (PMC6294072; doi:10.1016/j.stemcr.2018.10.008)
Supplement: Document S1. Supplemental Experimental Procedures, Figures S1–S7, and Tables S1 and S2 [file mmc1.pdf]

Stem Cell Reports, Volume *11*

Supplemental Information

Atrial-like Engineered Heart Tissue: An *In Vitro* Model of the Human Atrium

Marta Lemme, Bärbel M. Ulmer, Marc D. Lemoine, Antonia T.L. Zech, Frederik Flenner, Ursula Ravens, Hermann Reichenspurner, Miriam Rol-Garcia, Godfrey Smith, Arne Hansen, Torsten Christ, and Thomas Eschenhagen

Supplemental Information

Supplemental Data

Figure S1. Negative controls for flow cytometry, related to Figure 1B

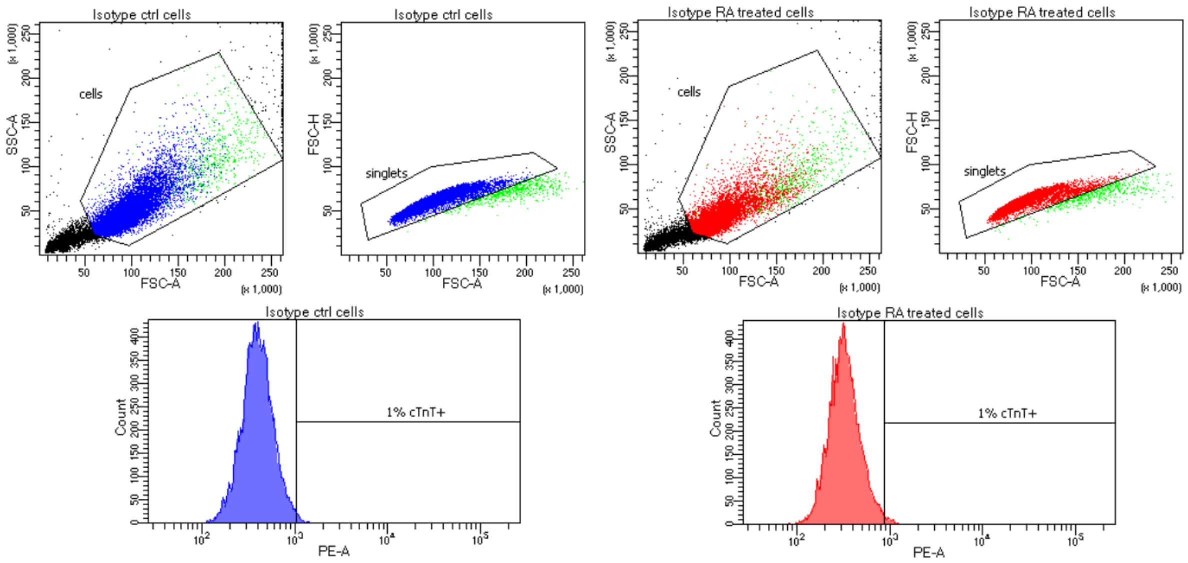

Figure S1. Flow cytometry negative controls of RA-treated and control hiPSC-CM using appropriate isotype antibodies.

Figure S2. Validation of WB antibodies, related to Figure 2B

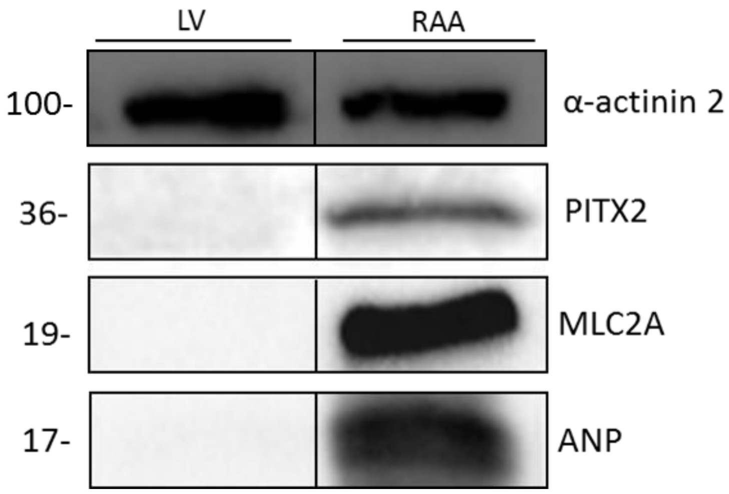

Figure S2. Validation of WB antibodies to test their specificity using human adult cardiac tissue (LV and RAA).

Figure S3. Experiment list for each cell line, related to Figure 2, 4, 5, 6, 7

	FACS	Cell area	qPCR	WB	EHT	IF	AP	TEM	Drug response	CCh: APD_{90} ↓	4-AP: APD_{20} ↑
C25	✓	✓	✓	✓	✓	✓	✓	✓	✓	7.9%±1.6% * n=6	38.3±1.9% ** n=6
ERC18	✓	✓	✓	×	✓	✓	✓	×	✓	20±2.6% * n=5	18.4±3.2% ** n=5
ERC1	✓	×	✓	×	✓	×	✓	×	✓	9.4±1.7% * n=5	21.2±1.8% ** n=5

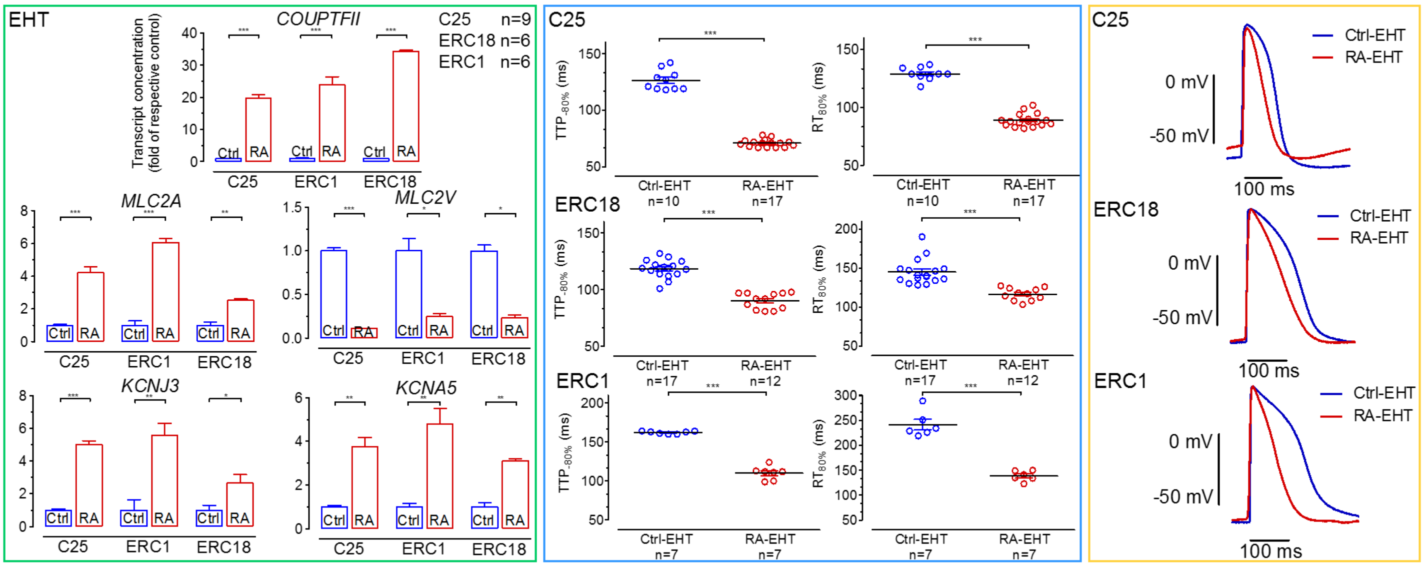

Figure S3. List of experiments performed on each cell line (C25, ERC18 and ERC1). For each cell line, EHT (C25 n=9, ERC1 n=6, ERC18 n=6) expression analysis of atrial specific genes (green box), contractility measurements (Ctrl/RA-EHT: C25 n=10/17, ERC18 n=17/12 and ERC1 n=7/7) (blue box) and AP recordings (yellow box) were performed. In the grey box EHT pharmacological response to atrial selective drug of each cell line (C25 n=6, ERC18 n=5, ERC1 n=5).
n values describe number of EHT obtained from 3 batches of C25 and 2 batches of ERC18 and ERC1. Error bars show mean±SEM.

Figure S4. COUPTFII staining, related to Figure 2 and 3

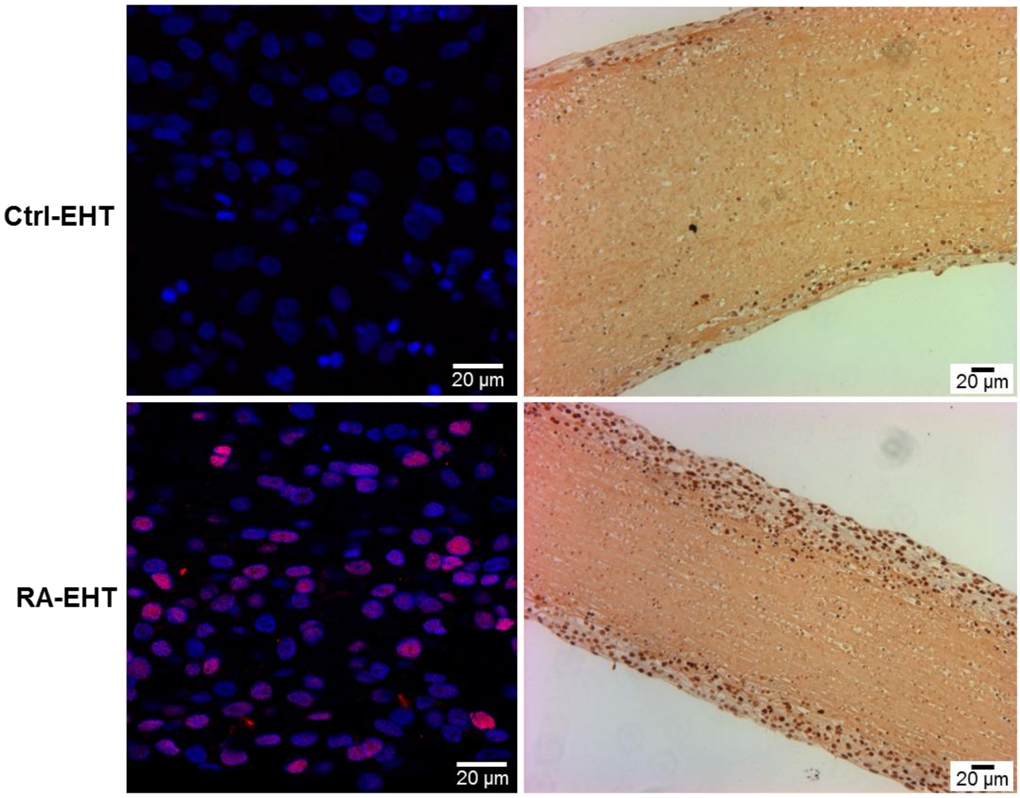

Figure S4. Immunofluorescence labeling (left) and immunohistochemistry (right) of COUPTFII expression in paraffin section of Ctrl- and RA-EHTs.

Figure S5. Live images of Ctrl- and RA-EHTs, related to Figure 3 and 4

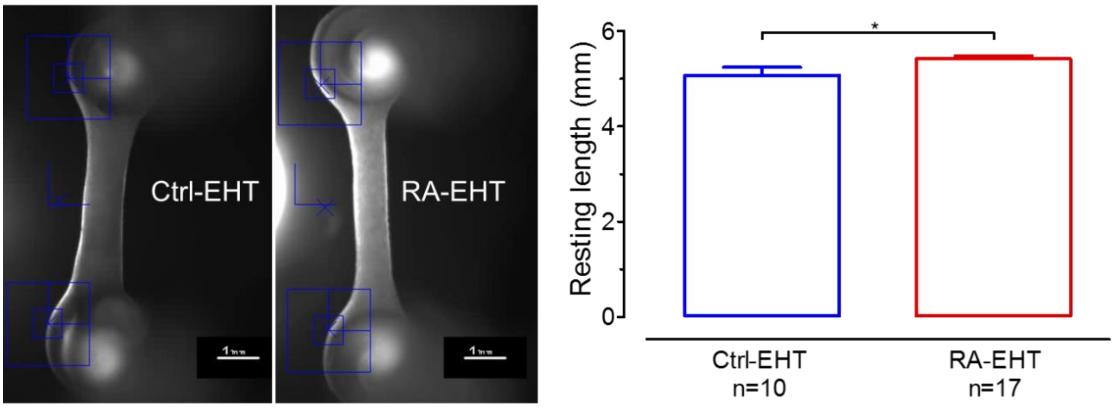

Figure S5. Live images of 20 day-old Ctrl- and RA-EHTs. Scale bar 1 mm. (B, right) RA-EHTs showed slightly higher resting length than Ctrl-EHTs (5.4±0.07 mm vs 5±0.16 mm, n=17/10 from 3 batches; p<0.05, unpaired t-test). Error bars show mean±SEM.

Figure S6. Cardiomyocyte organization in EHTs, related to Figure 3 and 4

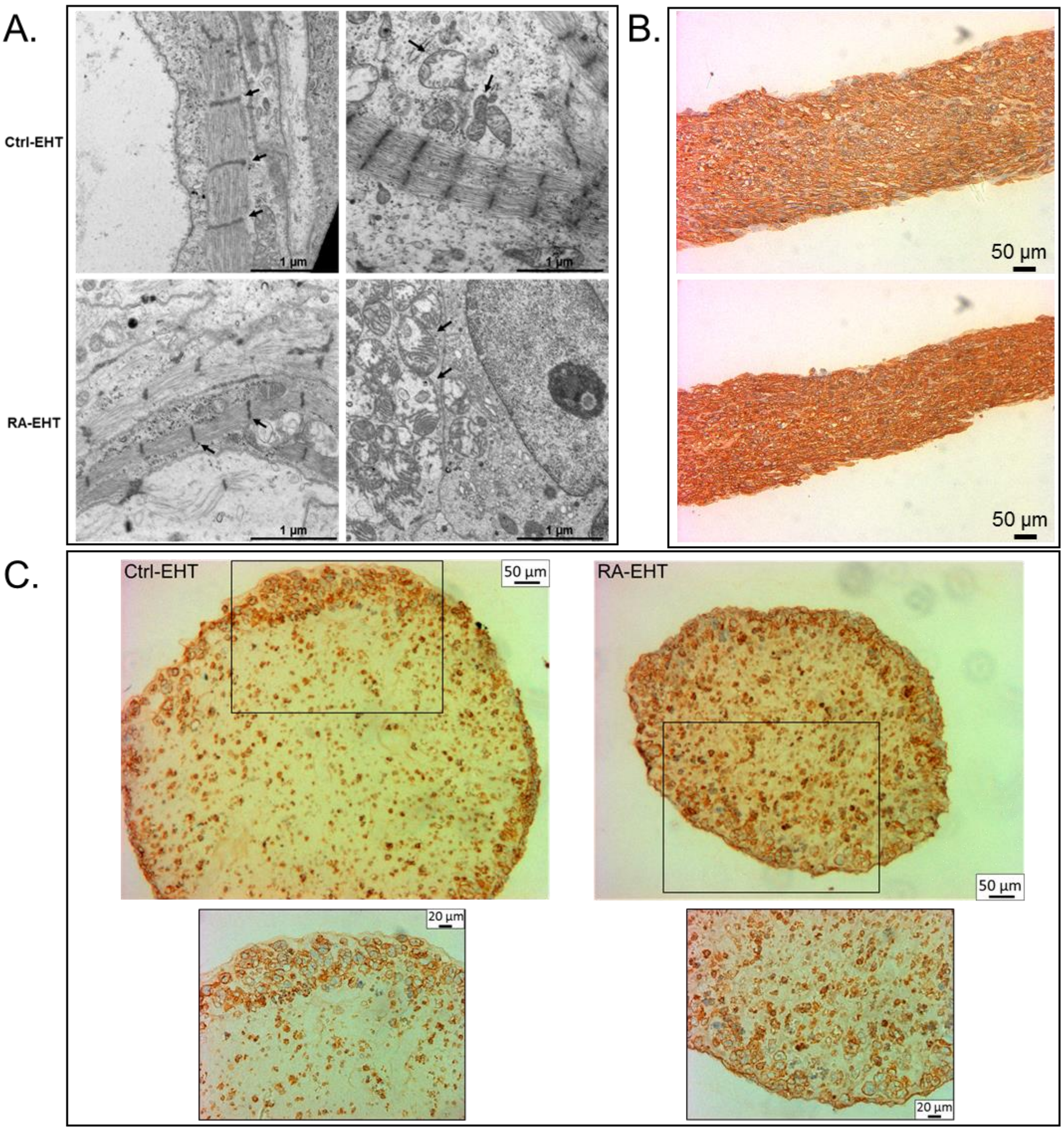

Figure S6. (A) Transmission electron microscopy of 20 day-old EHTs. The cytoplasm of both the Ctrl- and RA-EHTs contained well-developed sarcomeres. Qualitatively, RA-EHTs showed less organized sarcomeres than Ctrl-EHTs. The same finding was shown for fetal human atrial and ventricular cardiac muscle cells (Claycomb et al., 1989). Arrows indicate Z-discs and mitochondria. (B) Immunohistochemistry of α-actinin in paraffin sections of Ctrl- and RA-EHTs. (C) Immunohistochemistry of dystrophin in cross sections of Ctrl- and RA-EHTs.

Figure S7. Functional differences between Ctrl- and RA-EHTs, related to Figure 4, 5, 6 and 7

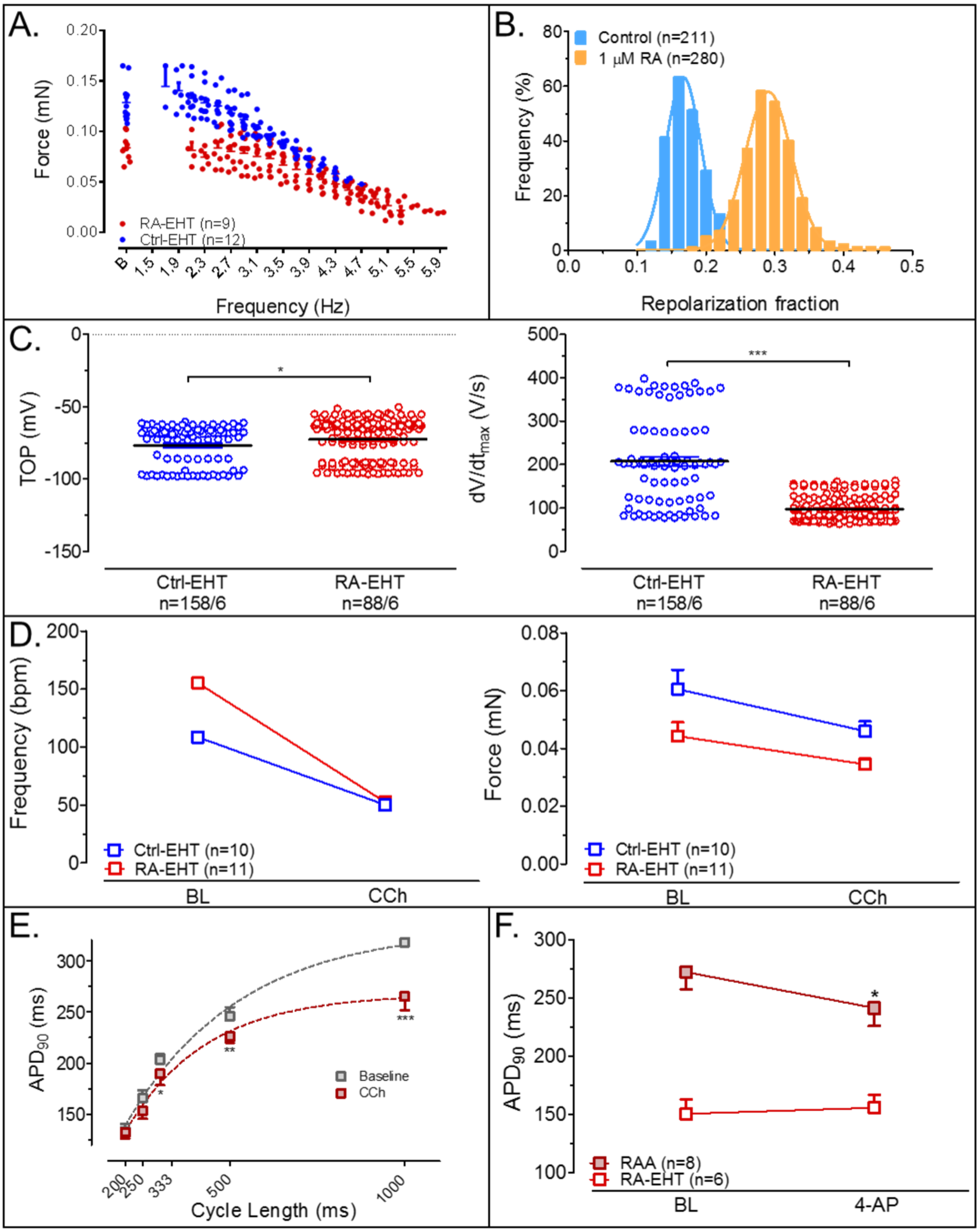

Figure S7. (A) Force-frequency relationship of RA-EHTs (n=9 from 2 batches) and Ctrl-EHTs (n=12 from 2 batches). (B) Frequency distributions for repolarization fraction of Ctrl (n=211 wells, 2 batches) and RA-treated cells (n=280 wells, 2 batches). (C) Take-off potential (left) and upstroke velocity (right) of RA-EHTs (n=88/6, number of impalements/EHTs, 3 batches) and Ctrl-EHTs (n=158/6, number of impalements/EHTs, 3 batches). RA-EHTs showed a less negative take-off potentials (-70±1.1 mV vs. -76±1.5 mV) with slower upstroke velocity (97.6±2.4 V/s vs. 207.6±10.6 V/s). (D) Beating frequency (left) and contractile force (right) of unpaced Ctrl- (n=10 from 3 batches) and RA-EHTs (n=11 from 3 batches) before (baseline, BL) and after CCh (10 µmol/L) exposure. (E) Rate-dependency of CCh-induced action potential shortening in adult human atrium (n=6 patients, $p<0.05$, paired t-test). An exponential function was fitted to the data points. (F) Effect of 4-AP (50 µmol/L) on APD_{90} in RAA (n=8 patients) and RA-EHTs (n=6 from 3 batches).
Error bars show mean±SEM.

2. Supplemental Tables

Table S1. List of RT-qPCR primers, related to Figure 2

Primer Name [HGNC gene name]		Primer Sequence [5'…3'] F: forward, R: reverse	Position CHR (GRCh37/hg19)	Ampl.-length [bp]	Primer T_m [°C]
COUPTFII	Chicken ovalbumin upstream promoter II	F: CCGAGTACAGCTGCCTCAA<br>R: TTTTCCTGCAAGCTTTCCAC	Chr15:96332648-96340955	91	59.41<br>57.11
COUPTFI	Chicken ovalbumin upstream promoter I	F: AAGCCATCGTGCTGTTCAC<br>R: GCTCCTCAGGTACTCCTCCA	Chr5:93586350-93595364	107	58.75<br>60.03
PITX2	Paired-like homeodomain transcription factor 2	F: AGCCATTCTTGCATAGCTCG<br>R: GTGTGGACCAACCTTACGGA	Chr4:110615342-110640316	109	58.41<br>59.61
IRX4	Iroquois Homeobox 4	F: TTGAGAGGTTAAGTTGGGGTTTG<br>R: CCACCCAATTTCTAAAACTAATTACC	Chr5:1878325-1878605	281	58.79<br>56.78
MLC2V	Myosin regulatory light chain 2, ventricular isoform	F: GATGTTCGCCGCCTTCCCCGC<br>R: GCAGCGAGCCCCCTCCTAGT	Chr12:110909841-110921578	106	68.75<br>66.01
MLC2A	Myosin regulatory light chain 2, atrial isoform	F: CACCGTCTTCCTCACACTCTT<br>R: AGGCACTCAGGATGGCTTC	Chr7:44138614-44141608	71	59.66<br>59.39
ANP	Atrial natriuretic peptide	F: ACAGGATTGGAGCCCAGAG<br>R: GGAGCCTCTTGCAGTCTGTC	Chr1:11845501-11847991	109	58.69<br>60.39
SLN	Sarcolipin	F: CTTGGTGTGCCCTCAGAAAT<br>R: TCAGTCAATCCCAGGACCAT	Chr11:107706906-107712530	179	58.08<br>58.03
KCNJ3	Potassium inwardly-rectifying channel, subfamily J, member 3	F: AAAAACGATGACCCCAAAGA<br>R: TGTCGTCATCCTAGAAGGCA	Chr2:154682604-154874329	98	55.46<br>58.15
KCNA5	Potassium voltage-gated channel subfamily A member 5	F: CGAGGATGAGGGCTTCATTA<br>R: CTGAACTCAGGCAGGGTCTC	Chr12:5043632-5047075	186	56.79<br>59.75
SK2	Small conductance calcium-activated potassium channel 2	F: ATGAGCAGCTGCAGGTACAA<br>R: CTAGCTACTCTCTGATGAGG	Chr5:114011956-114540550	1740	59.67<br>53.52
SK3	Small conductance calcium-activated potassium channel 3	F: GTTCTTTCACCCCCTCTTCTTTC<br>R: TTGGCTTGCTTCGGTTCTCT	Chr1:154680180-154887560	123	59.18<br>59.89
GUSB	β-Glucuronidase	F: AAACGATTGCAGGGTTTCAC<br>R: CTCTCGTCGGTGACTGTTCA	Chr2:140043116-142302796	171	57.21<br>59.41

Table S1. Primer sequences used for RT-qPCR assays.

Table S2. Clinical information of patients, related to Figure 5, 6 and 7

	RAA	LV
N	38	30
Gender [m/f]	20 / 18	12 / 18
Age [years]	70.2 ± 2.3	59.4 ± 3.1
BMI [kg/m^2]	26.3 ± 0.8	25.3 ± 0.7
Sinus rhythm	38	30
Hypertension, n	38	16
Diabetes mellitus, n	5	5
Hyperlipidaemia, n	12	7
Coronary artery disease, n	21	3
Valve disease, n	17	20
Hypertrophic obstructive cardiomyopathy, n	0	5
LVEF [%]	59.3 ± 3.3	52.4 ± 3.4
Cardiovascular medication (n)		
Digitalis	2	1
ACE-Inhibitors	9	16
AT_1-blockers	2	5
β-blockers	12	17
Ca^{2+}-channel-blockers	6	1
Diuretics	8	12
Nitrates, n	0	1
Lipid-lowering drugs	11	8

Table S2. Patient characteristics belonging to the atrial and ventricular tissue used for experiments. Abbreviations: AT, angiotensin receptor; LVEF, left ventricular ejection fraction. Mean±SEM.

3. Supplemental Experimental procedures

3.1 Cell size measurement

As previously published (Prondzynski et al., 2017) for cell size analysis, confocal microscopy Zeiss LSM 800 with Airyscan technology was used. HiPSC-CMs were stained for α-actinin 2 (1:800, Sigma) in 96-well plates and >100 images per sample were recorded. Cell sizes from confocal images were measured by using Fiji software (ImageJ). Quality criteria for hiPSC-CM inclusion were set for single cells with well-formed sarcomeres.

3.2 Flow cytometry

For determination of differentiation efficiency, 2 x 10^5 cells were subjected to flow cytometry analysis. Cells were fixed in cold methanol (−20 °C) for 20 min at 4 °C (Breckwoldt et al., 2017) and permeabilized in permealization buffer containing 5% fetal bovine serum, 0.5% saponin (Sigma) ad 0.05% sodium azide. For intracellular staining, cells were stained with directly labeled antibody anti-cardiac Troponin T-FITC, 1:10 dilution (Miltenyi Biotec) in permeabilization buffer for 30 min at 4 °C. As negative control we used appropriate isotype antibody (Figure S1). Stained cells were analyzed using BD FACSCanto II Flow Cytometer and the BD FACSDiva Software 6.0.

3.3 Gene expression analysis

Total RNA was extracted from hiPSC-CMs, cultured for 14-20 days either in ML or EHT format. Extraction of total RNA was performed with RNeasy Mini Kit (Qiagen) according to manufactures instructions. For EHTs, proteinase K (Thermo Scientific) digestion was performed before extraction. Real-time qPCR experiments were performed on three independent Ctrl- and RA-ML/EHT generations. For assessing gene expression by qPCR, cDNA was synthesized from approximately 200 ng of total RNA. RNA was reverse transcribed into cDNA using high capacity cDNA reverse transcription kit (Applied Biosystems). QPCR was performed using Maxima SYBR Green/ROX (Thermo Scientific) on an ABI Prism instrument (Applied Biosystems). Each reaction was performed in triplicates and non-template reaction (replacing cDNA with water) was used as negative control. The cycling parameters were 50 °C for 2 min followed by 95 °C for 10 min, 15 seconds at 95 °C and 1 minute at 60 °C for 40 cycles. mRNA-specific CT values were normalized with CT values for human *GUSB* (beta glucuronidase, housekeeping gene) and with their respective controls (Ctrl-ML and Ctrl-EHT). Relative differences between RA and Ctrl samples were calculated with ΔΔCt method for relative quantifications. Primer sequences are enclosed in the Table S1. Candidates markers for atrial and ventricular phenotype were chosen based on previous publications (Ellinghaus et al., 2005; Gaborit et al., 2007; Wobus et al., 1995).

3.4 Protein analysis by Western Blot

For analysis of proteins, ML were detached with trypsin (3 min, 37°C), spun down (100 g, 5 min) and subsequently dissolved in 100 µL T-PER Tissue Protein Extraction Reagent (ThermoScientific, 78510) with cOmplete Mini EDTA-free protease inhibitor cocktail (Roche Diagnostics, 04693159001). To 10 µl ML lysate, 1xlaemmli buffer was added, subsequently the mixture was heated for 5 min at 95 °C. Proteins were separated by 12% acrylamide/bisacrylamide (29:1, BioRad, 1610156) gels and thereafter transferred onto nitrocellulose (NC) or polyvinylidene fluoride (PVDF) membranes (0.45 µm). Washing steps were performed with TBS-Tween 0.1%, blocking with 5% skim milk powder. Primary antibodies were incubated ON at 4°C in TBS-Tween 0.1%, secondary antibodies for 1 hour at RT in 5% skim milk powder/TBS-Tween 0.1%. Following primary antibodies were used: ANP (NC; 1:10000; Abcam, ab91250), MLC2A (NC; 1:1000; Synaptic Systems, 311011), PITX-2 (NC; 1:250; Invitrogen, PA5-11479), COUPTFII (PVDF; 1:5000; Perseus Proteomics, PP-H7147-00). Following secondary antibodies were used: α-rabbit IgG peroxidase-conjugate (1:10 000; Sigma, A9044), α-mouse IgG peroxidase-conjugate (1:10 000; Sigma, A3682). Visualization was performed with the Clarity Western ECL Substrate (BioRad) at the ChemiDoc imaging system (BioRad).

3.5 Immunohistochemistry and immunofluorescence

14-20 day-old EHTs were fixed in formaldehyde (Roti®-Histofix 4%, Carl Roth, P087.3) ON at 4 °C. After embedding in paraffin, 4 µm thick longitudinal sections were processed for immunohistochemical staining (monoclonal mouse anti-α-actinin 1:800, Sigma A7811 monoclonal rabbit anti-MLC2v 1:200, ProteintecTM, 10906-1-AP; monoclonal mouse anti-MLC2A 1:200, Synaptic systems 311011, monoclonal mouse anti-COUPTFII 1:200; Perseus Proteomics, PP-H7147-00; monoclonal mouse anti-dystrophin 1:200, Millipore MAB1645). All microscopic images were taken on an Axioskop 2 microscope (Zeiss).

For whole mount immunofluorescence staining, fixed EHTs were blocked (6 h in TBS 0.05 M pH 7.4, 10% FCS, 1% BSA, 0.5% Triton X-100), incubated in antibody solution (TBS 0.05 M pH 7.4, 1% BSA, 0.5% Triton X-100) with primary antibodies ON (monoclonal rabbit anti-MLC2v 1:200, ProteintecTM, 10906-1-AP; monoclonal mouse anti-MLC2A 1:200, Synaptic systems 311011; monoclonal mouse anti-COUPTFII 1:200; Perseus Proteomics, PP-H7147-00), washed repeatedly with PBS, incubated in antibody solution with secondary antibodies and other stainings for two hours at RT (Alexa Fluor® 488 goat-anti-rabbit 1:800, Invitrogen; Alexa Fluor® 546 goat-anti-mouse 1:800; DAPI Sigma Aldrich D9564 1:1000), rinsed 2-3 times in PBS and embedded in Fluoromount-G® (SouthernBiotech, 0100-01) in microscope slides (Carl Roth, H884.1). MLs were cultivated for 14 days and then fixed for 20 min at 4 °C and stained accordingly with the exception of using a different permeabilization buffer (1x PBS, milk powder 3% (w/v), Triton X-100). Immunofluorescence images were acquired using Zeiss LSM 800.

3.6 Drugs

CCh and 4-AP were obtained from Sigma-Aldrich (St. Louis, MO, USA). CCh, a muscarinic receptor agonist, was prepared as a 10 mmol/L stock solution in DMSO and stored at -20 ºC. 4-AP was prepared as a 1 mmol/L stock solution in Tyrode's solution, pH adjusted to 7.4 and stored at 4 ºC. All stock solutions were diluted appropriately before use.

3.7 Transmission electron microscopy

For transmission electron microscopy (TEM), EHTs were washed twice in PBS and incubated in 2-butandionemonoxime (Sigma, B0753; 30 mM in PBS, 10 min, 37 °C) to relax sarcomeres and fixed overnight in glutaraldehyde (0.36%, pH 7.0-7.5, 4 °C). Fixed EHTs were removed from silicone racks and subjected to postfixation in osmium tetroxide solution (1%, 2 h; Science Services, 19110), dehydration and embedding in a glycidether-based resin. Ultra-thin sections (50 nm) were prepared and analyzed on a Zeiss LEO 912AB.

3.8 Force-frequency relationship

Force-frequency relationship was measured on 14 day-old EHTs in Gibco DMEM, High Glucose (LS11965092) supplemented with 25 mmol/L HEPES and 1.8 mmol/L $CaCl_2$ pre-equilibrated overnight (37 °C, 7% CO_2, 40% O_2). Following a stabilization period of 30 min in the new medium, contractile force of EHTs under electrical pacing was measured. Spontaneous contraction activity was recorded to determine the starting frequency of the force-frequency relationship. The electrical pacing was performed using a biphasic stimulation characterized by 2.5 V and 4 ms duration. EHT contraction force was recorded for 10 seconds at each frequency. Pacing frequency was varied from 1.5 to 6 Hz in 0.2 Hz steps.

3.9. Flow volumetry analysis

As recently published (Mosqueira et al., 2018) flow volumetry analysis was performed to estimate the volume of hiPSC-CMs after differentiation and after 14 days of EHT culture. In order to establish a calibration curve relating forward light scattered and cell size, calibration beads of known dimensions (2-14.3µm diameter, Spherotech #PPS-6K) were analysed using BD FACSCanto II Flow Cytometer, after excluding debris and duplets/triplets. Only the volume of cardiac troponin T positive cells was measured. Therefore, cell size of hiPSC-CMs was estimated as the volume of a perfect sphere ($V = \frac{\pi}{6} * d^3$) using the calibration curve.

4 Experimental protocol of human samples

Human tissue samples were transported in Ca^{2+}-free transport solution at 20–25 °C for maximum 30 min, composition in mmol/L: 100.0 NaCl, 10.0 KCl, 1.2 KH_2PO_4, 5.0 $MgSO_4$, 50 taurin, 5 MOPS, 30 butanedione monoxime (BDM), pH 7.0. Before the start of each measurement, tissue samples were superfused with Tyrode's solution at 36.5±0.5 °C for at least 30 minutes.

4. Supplemental References

Breckwoldt, K., Letuffe-Brenière, D., Mannhardt, I., Schulze, T., Ulmer, B., Werner, T., Benzin, A., Klampe, B., Reinsch, M.C., Laufer, S., et al. (2017). Differentiation of cardiomyocytes and generation of human engineered heart tissue. Nat. Protoc. *12*, 1177–1197.

Claycomb, W.C., Delcarpio, J.B., Guice, S.E., and Moses, R.L. (1989). Culture and characterization of fetal human atrial and ventricular cardiac muscle cells. Vitr. Cell. Dev. Biol. Tissue Cult. Assoc. *25*, 1114–1120.

Ellinghaus, P., Scheubel, R.J., Dobrev, D., Ravens, U., Holtz, J., Huetter, J., Nielsch, U., and Morawietz, H. (2005). Comparing the global mRNA expression profile of human atrial and ventricular myocardium with high-density oligonucleotide arrays. J. Thorac. Cardiovasc. Surg. *129*, 1383–1390.

Gaborit, N., Le Bouter, S., Szuts, V., Varro, A., Escande, D., Nattel, S., and Demolombe, S. (2007). Regional and tissue specific transcript signatures of ion channel genes in the non-diseased human heart. J. Physiol. *582.2*, 675–693.

Mosqueira, D., Mannhardt, I., Bhagwan, J.R., Lis-Slimak, K., Katili, P., Scott, E., Hassan, M., Prondzynski, M., Harmer, S.C., Tinker, A., et al. (2018). CRISPR/Cas9 editing in human pluripotent stem cell-cardiomyocytes highlights arrhythmias, hypocontractility, and energy depletion as potential therapeutic targets for hypertrophic cardiomyopathy. Eur. Heart J. *0*, 1–16.

Prondzynski, M., Krämer, E., Laufer, S.D., Shibamiya, A., Pless, O., Flenner, F., Müller, O.J., Münch, J., Redwood, C., Hansen, A., et al. (2017). Evaluation of MYBPC3 trans-Splicing and Gene Replacement as Therapeutic Options in Human iPSC-Derived Cardiomyocytes. Mol. Ther. - Nucleic Acids *7*, 475–486.

Wobus, A.M., Rohwedel, J., Maltsev, V., and Hescheler, J. (1995). Development of Cardiomyocytes Expressing Cardiac-Specific Genes, Action Potentials, and Ionic Channels during Embryonic Stem Cell-Derived Cardiogenesis. Ann. N. Y. Acad. Sci. *752*, 460–469.
