# Supplementary material for: Atrial-like Engineered Heart Tissue: An In Vitro Model of the Human Atrium
Source: Stem Cell Reports. 2018 Nov 8;11(6):1378–90. doi: 10.1016/j.stemcr.2018.10.008 (PMC6294072; doi:10.1016/j.stemcr.2018.10.008)
Supplement: Document S2. Article plus Supplemental Information [file mmc4.pdf]

Stem Cell Reports
Article

OPEN ACCESS

Atrial-like Engineered Heart Tissue: An *In Vitro* Model of the Human Atrium

Marta Lemme,[1,2,3] Bärbel M. Ulmer,[1,2] Marc D. Lemoine,[1,2,7] Antonia T.L. Zech,[1,2] Frederik Flenner,[1,2] Ursula Ravens,[4,6] Hermann Reichenspurner,[2,5] Miriam Rol-Garcia,[3] Godfrey Smith,[3] Arne Hansen,[1,2] Torsten Christ,[1,2] and Thomas Eschenhagen[1,2,*]
[1]Institute of Experimental Pharmacology and Toxicology, University Medical Center Hamburg-Eppendorf, Martinistrasse 52, 20246 Hamburg, Germany
[2]DZHK (German Centre for Cardiovascular Research), Partner Site Hamburg/Kiel/Lübeck, 20246 Hamburg, Germany
[3]Clyde Biosciences Ltd, BioCity Scotland, Bo'Ness Road, Newhouse, Lanarkshire ML1 5UH, UK
[4]Institute of Experimental Cardiovascular Medicine, University Heart Center Freiburg-Bad Krozingen, 79106 Freiburg, Germany
[5]Department of Cardiovascular Surgery, University Heart Center, 20246 Hamburg, Germany
[6]Institute of Physiology, Medical Faculty Carl Gustav Carus, TU Dresden, 01307 Dresden, Germany
[7]Department of Cardiology-Electrophysiology, University Heart Center, 20246 Hamburg, Germany
*Correspondence: t.eschenhagen@uke.de
https://doi.org/10.1016/j.stemcr.2018.10.008

SUMMARY

Cardiomyocytes (CMs) generated from human induced pluripotent stem cells (hiPSCs) are under investigation for their suitability as human models in preclinical drug development. Antiarrhythmic drug development focuses on atrial biology for the treatment of atrial fibrillation. Here we used recent retinoic acid-based protocols to generate atrial CMs from hiPSCs and establish right atrial engineered heart tissue (RA-EHT) as a 3D model of human atrium. EHT from standard protocol-derived hiPSC-CMs (Ctrl-EHT) and intact human muscle strips served as comparators. RA-EHT exhibited higher mRNA and protein concentrations of atrial-selective markers, faster contraction kinetics, lower force generation, shorter action potential duration, and higher repolarization fraction than Ctrl-EHTs. In addition, RA-EHTs but not Ctrl-EHTs responded to pharmacological manipulation of atrial-selective potassium currents. RA- and Ctrl-EHTs' behavior reflected differences between human atrial and ventricular muscle preparations. Taken together, RA-EHT is a model of human atrium that may be useful in preclinical drug screening.

INTRODUCTION

More than 33 million people worldwide suffer from atrial fibrillation (AF), with increasing prevalence (Chugh et al., 2014). Uncoordinated high-frequency contractions in the atria lead to atrial stunning, increasing the risk for blood clots, stroke, and heart failure (Marini et al., 2005; Wang et al., 2003). Pulmonary vein isolation by catheter ablation and antiarrhythmic drugs represent two treatment options. While ablation is not always effective, particularly in advanced forms of the disease, the available antiarrhythmic drugs have limited efficacy and cause adverse effects (Schotten et al., 2011).

Drug development is hampered by the difficulty in isolating and maintaining human atrial cardiomyocytes (CMs). Animal models do not accurately represent the physiology of human CMs, limiting their predictive power (Denayer et al., 2014). CMs generated from human induced pluripotent stem cells (hiPSCs) may offer a platform to study disease mechanism and evaluate novel drugs. However, hiPSC-CMs are believed to consist predominantly of ventricular-like cells with a small percentage of atrial-like and nodal-like cells (van den Berg et al., 2016; Blazeski et al., 2012; Ma et al., 2011; Marczenke et al., 2017). Recent developments therefore aim at establishing hiPSC-derived models of predominantly atrial-like myocytes.

Retinoids regulate heart morphogenesis and contribute to cardiac reprograming. Specification of cardiac progenitors can be altered by all-*trans* retinoic acid (RA) treatment (Zaffran et al., 2014). Previous studies have shown that treatment of human embryonic stem cell (hESC) and hiPSC differentiation cultures with RA is sufficient to generate cells that display electrophysiological properties and gene expression patterns characteristic of early atrial-like myocytes (Chen et al., 2017; Cyganek et al., 2018; Devalla et al., 2015; Lee et al., 2017; Josowitz et al., 2014; Zhang et al., 2011).

The current study was conducted to evaluate the suitability of hiPSC-derived atrial-like myocytes in engineered heart tissue (EHT) format (Mannhardt et al., 2016). This system offers a more physiological cell environment and allows the monitoring of the major parameters of heart function: force, pacemaking activity, contraction and relaxation kinetics (Hansen et al., 2010; Mannhardt et al., 2016), as well as standard cardiac electrophysiology (Lemoine et al., 2017).

RESULTS

RA Treatment Decreases Cell Size

We used RA to induce an atrial phenotype in hiPSC-CMs. HiPSC-CMs were differentiated following an established three-step protocol (Breckwoldt et al., 2017, Figure 1A). RA treatment (1 μmol/L) between day 4 and day 7 after mesodermal induction did not significantly alter the

This is an open access article under the CC BY license (http://creativecommons.org/licenses/by/4.0/).

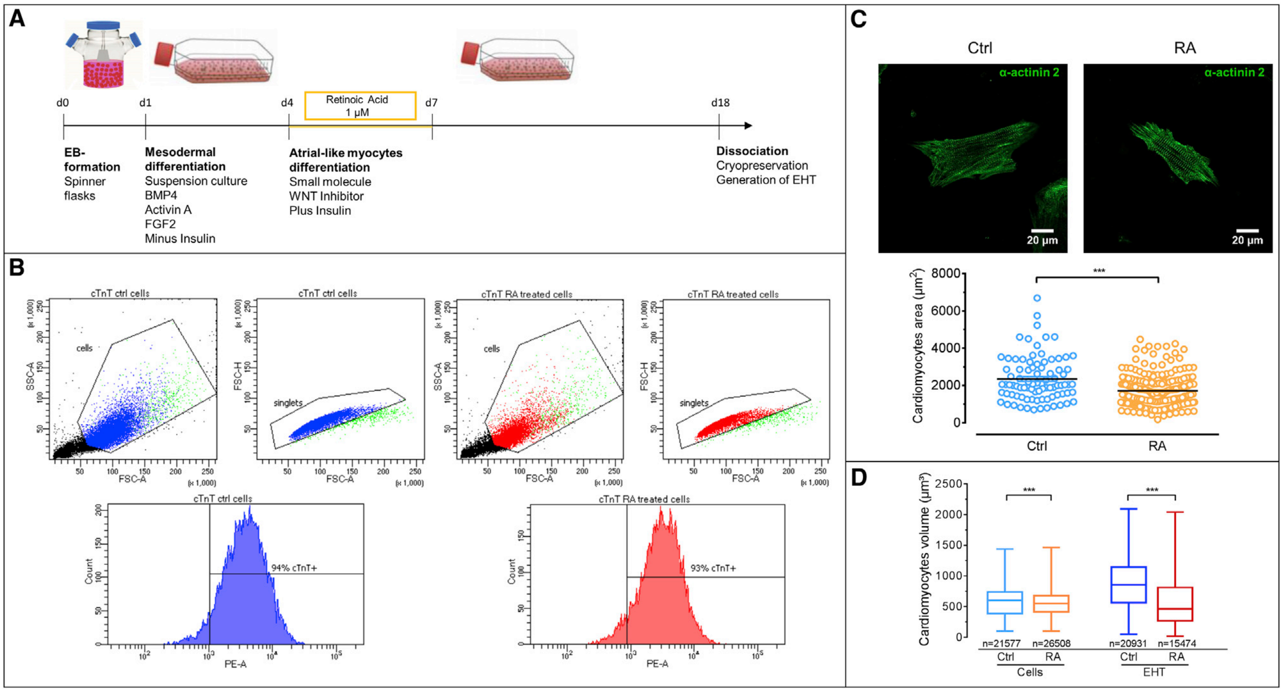

Figure 1. Effect of RA on Cardiac Differentiation Protocol

(A) EB-based cardiac differentiation protocol. RA (1 μmol/L) was added from day 4 to day 7 to induce differentiation toward an atrial-like phenotype.

(B) Flow cytometry analysis of RA-treated and Ctrl cells.

(C) Analysis of cell area using α-actinin 2 staining. RA-treated cells show smaller area than Ctrl cells (1,736.2 ± 64.4 μm^2 versus 2,468.7 ± 192 μm^2, n = 209 and 88 from three batches each; $p < 0.05$, unpaired t test).

(D) Box plots showing median, first, and third quartile of the volume of Ctrl versus RA-treated CMs. (Left) Volume of RA-treated cells after differentiation was smaller than Ctrl cells (560 ± 1.3 μm^3 versus 588 ± 1.6 μm^3, n = 26,508 and 21,577 from three batches each; $p < 0.05$, unpaired t test). (Right) Volume of cells dissociated from RA-EHTs was smaller than from Ctrl-EHTs (569 ± 3 μm^3 versus 854 ± 2.8 μm^3, n = 15,474 and 20,931 from 4 EHTs each; $p < 0.05$, unpaired t test).

Error bars show means ± SEM.

percentage of cardiac troponin T (cTnT)-positive cells (Figures 1B and S1), indicating no effect on principal cardiac differentiation efficiency. However, cell area as a surrogate of cell size was smaller in RA-treated cells (Figure 1C). While substantial overlap was noted between RA-treated and control hiPSC-CMs, mean values were significantly smaller in RA (1736 ± 64 μm^2 versus 2469 ± 192 μm^2, n = 209 and 88; $p < 0.05$, unpaired t test).

Cell size was also estimated as the volume of a perfect sphere using flow volumetry analysis (Mosqueira et al., 2018) (Figure 1D) after hiPSC-CM differentiation and after 14 days of EHT culture. Volume of RA-treated cells was smaller than control (Ctrl) cells (560 ± 1.3 μm^3 versus 588 ± 1.6 μm^3, n = 26,508 and 21,577; $p < 0.05$, unpaired t test), reflecting the smaller size of native atrial compared with ventricular CMs, although at an overall lower level (Bensley et al., 2016; Claycomb et al., 1989). After 14 days of EHT culture, the difference in volume of RA-treated cells versus Ctrl cells increased, due to an increase in size in Ctrl-EHTs (569 ± 3 μm^3 versus 854 ± 2.8 μm^3, n = 15,474 and 20,931; $p < 0.05$, unpaired t test).

RA Treatment Increases Gene Expression and Protein Level of Atrial-Specific Markers

Several proteins are differentially expressed between atrial and ventricular myocardium and can therefore be used as discriminators (Ellinghaus et al., 2005; Gaborit et al., 2007; Wobus et al., 1995) (Table S1). We used RT-qPCR to quantify transcript concentrations of the corresponding genes in monolayer cultures (MLs) and EHTs. Housekeeping genes *GUSB* and *CTNT* did not differ between Ctrl-MLs and Ctrl-EHTs. RA treatment consistently decreased mRNA levels of the ventricular markers *IRX4* and *MLC2V* (for full gene names see Table S1), whereas an increase was observed for atrial transcription factors (*COUPTFII*, *COUPTFI*, *PITX2*), atrial markers (*MLC2A*, *ANP*, *SLN*), and atrial-specific ion channels (*KCNJ3*, *KCNA5*, *SK2*, and *SK3*), both in MLs and EHTs (Figure 2A).

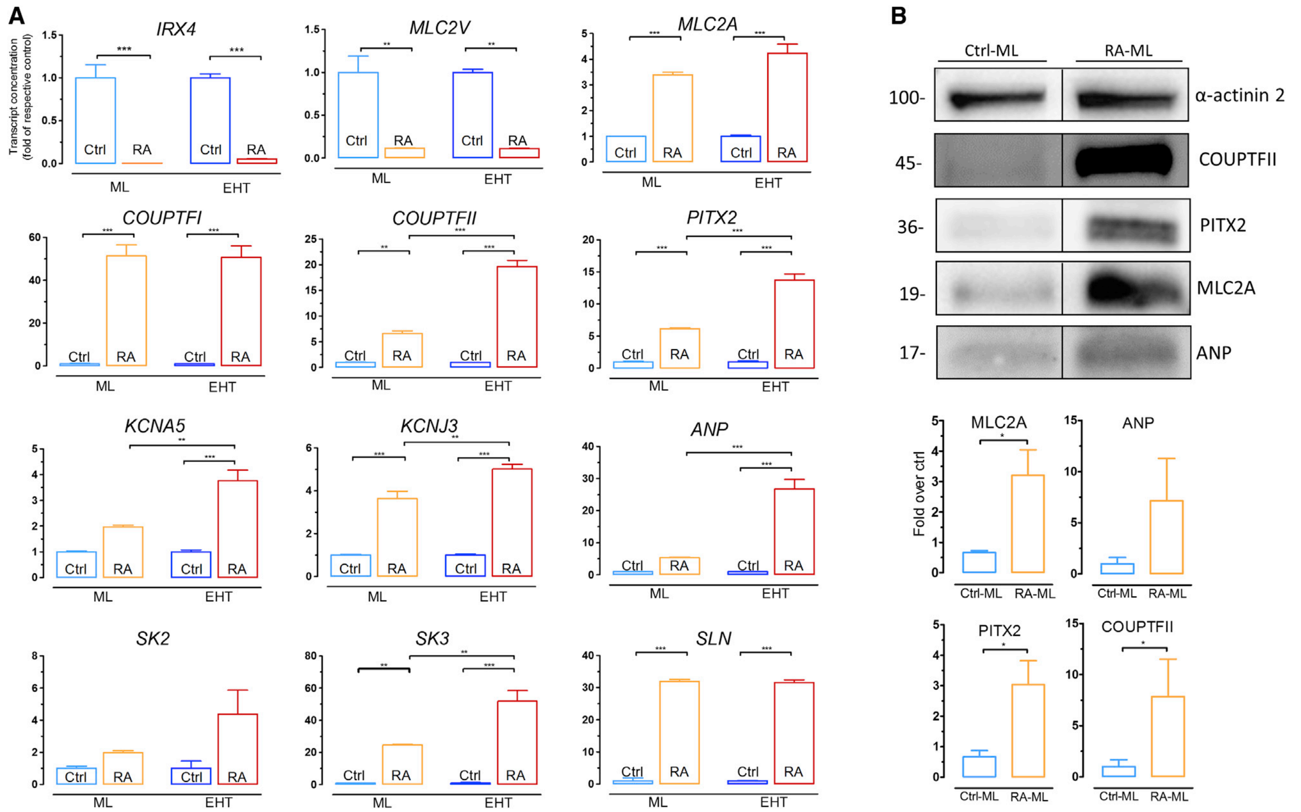

Figure 2. RA Treatment of hiPSCs Promotes Expression of Atrial-Specific Genes

(A) RT-qPCR of selected genes at day 14 to validate upregulation of atrial and downregulation of ventricular markers in RA-MLs/EHTs (n = 9 from three batches each) compared with Ctrl-MLs/EHTs (n = 9 from three batches each). Cycle treshold (CT) values were normalized with CT values for human *GUSB*. Transcript concentrations are shown in the graph as folds of their respective Ctrl.

(B) Western blotting of 14-day-old MLs with antibodies against α-actinin 2, COUPTFII, PITX2, MLC2A, and ANP (n = 3 batches).

Error bars show means ± SEM.

In six out of ten atrial genes, RA-induced increase in expression was larger in EHTs than in MLs (Figure 2A), suggesting a further atrial specification in a 3D model of auxotonically beating EHT. The higher mRNA expression of *MLC2A*, *ANP*, *PITX2*, and *COUPTFII* in RA-MLs compared with Ctrl-MLs was confirmed on the protein level (Figures 2B, S2, and S4). These data suggest that RA induces atrial specification in hiPSC-CMs.

RA Treatment Reduces the Number of MLC2V$^+$ and Increases that of MLC2A$^+$ Cells

EHTs were casted from Ctrl- and RA-treated hiPSC-CMs with a percentage of cTnT$^+$ cells between 75% and 95%. EHT generation from Ctrl- and RA-treated hiPSC-CMs was highly reproducible with more than 90% of EHTs forming functional contracting tissues. RA-EHTs showed slower development than Ctrl-EHTs, indicated by a later onset of spontaneous beating. At steady state (~day 20), the resting length of RA-EHTs was higher than for Ctrl-EHTs (5.4 ± 0.07 mm versus 5.0 ± 0.16 mm; $p < 0.05$, unpaired t test; Figure S5). Ctrl- and RA-EHTs showed a dense network of α-actinin-positive myocytes (Figure S6B). CMs in EHTs showed predominant longitudinal orientation along force lines and well-developed sarcomeric organization (Figures S6A and S6B). Dystrophin staining of CMs in cross sections (Figure S6C) showed that the majority of the cells in EHTs were concentrated in the outer layers near the surface. This observation has been made previously (Hirt et al., 2014; Vollert et al., 2013) and is likely due to better nutrient and oxygen supply and the stronger force lines at the edges.

Ctrl- and RA-EHTs could be easily distinguished by the different expression of myosin light chain isoforms. RA-treated CMs gave rise to MLs and EHTs characterized by a high fraction of MLC2A$^+$ cells and low fraction of MLC2V$^+$ cells, while Ctrl MLs and EHTs displayed the opposite distribution (Figure 3). Human adult heart tissues showed almost exclusive expression of MLC2A in specimens from right atrial appendages (RAAs) and of MLC2V in specimens from left ventricles (LVs) (Figure 3B). These findings support the conclusion that RA signaling

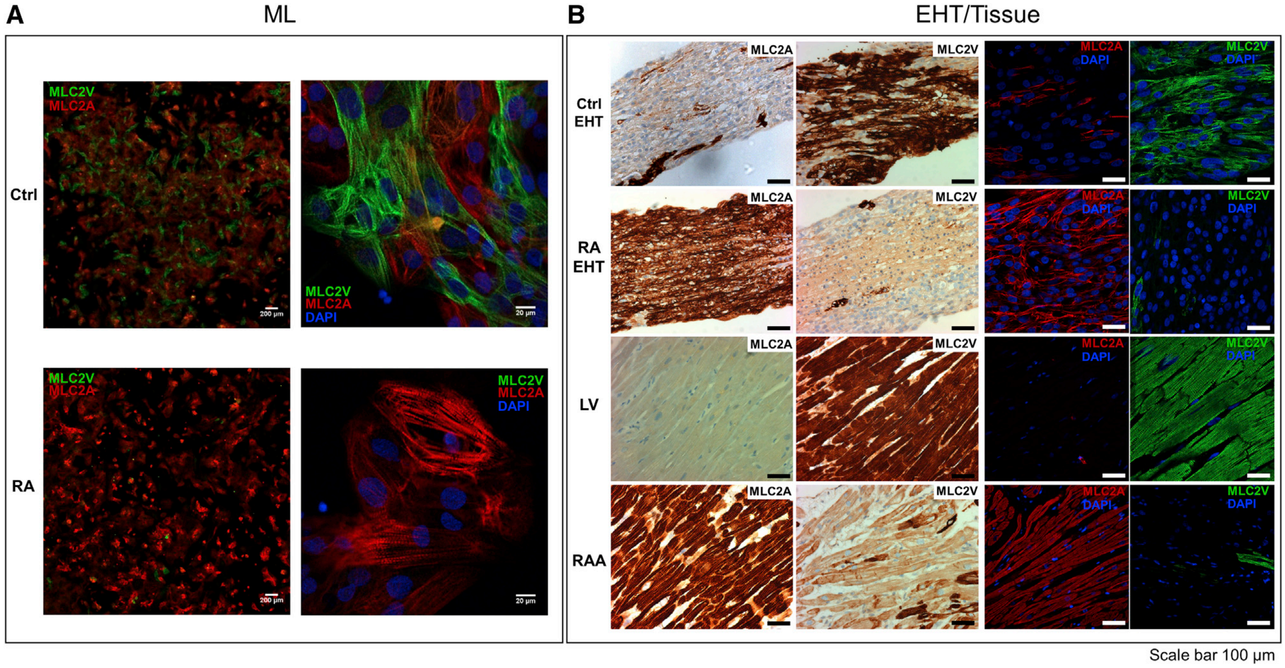

Figure 3. RA Treatment Shifts Expression of MLC from MLC2V to MLC2A

(A) Immunofluorescence labeling of 14-day-old MLs. 2.5× (left) and 40× (right) magnification of Ctrl and RA-MLs. Merged staining of MLC2V (green), MLC2A (red), and DAPI (blue).

(B) Immunohistochemistry of MLC2V and MLC2A expression in EHTs and human tissue (first two columns). Immunofluorescence of MLC2V (green), MLC2A (red), and DAPI (blue) (last two columns). Scale bar for all images represents 100 μm. Please note that the images show similar areas of evenly distributed CM network throughout the diameter of EHTs while generally the majority of the cells are located at the outer layer of the EHT (Figure S6C; Hirt et al., 2014; Vollert et al., 2013).

promotes the development of atrial-like myocytes at the expense of the ventricular phenotype.

RA Treatment Increases Beating Frequency and Speeds up Contraction Kinetics

EHTs beat faster than MLs, independently of the presence or absence of RA during differentiation. RA treatment significantly increased beating frequency in both culture formats (Figure 4), but its effect compared with Ctrl protocol was larger in EHTs than in MLs (increase by 61% ± 4% versus 28% ± 2%; $p < 0.05$, unpaired t test). Force was lower in EHTs from RA-treated hiPSC-CMs (RA-EHTs) than those from Ctrl-EHTs (Figure 4B). Contraction amplitudes as calculated by the MUSCLEMOTION algorithm (Sala et al., 2018) are arbitrary measures that cannot be used to distinguish absolute level of ML contractility. Ctrl-EHTs showed an overall inverse force-frequency relationship between 1.7 and 4.7 Hz. RA-EHTs showed a flat relation between 2.1 and 2.9 Hz and then a similarly inverse force-frequency relationship up to 5.9 Hz (Figure S7A). Since contraction kinetics are faster in RAA than LV (Berk et al., 2016; Molenaar et al., 2013), we analyzed time to peak (TTP) and relaxation time (RT). RA treatment accelerated contraction kinetics in both ML and EHT culture conditions (Figures 4A and 4B, Videos S1 and S2). To rule out that differences in contraction kinetics were the consequence of different spontaneous beating frequencies, kinetics were also analyzed under frequency Ctrl (3 Hz). Under these conditions, TTP and RT were 80 ± 0.65 ms and 117 ± 1.8 ms for RA-EHTs (n = 6) and 110 ± 1.3 ms and 123 ± 2.5 ms for Ctrl-EHTs (n = 5), respectively. Thus, the observed differences between RA- and Ctrl-EHTs were not due to differences in baseline frequency.

These functional data show that RA treatment changes contractility pattern toward an atrial-like phenotype.

RA Treatment Reduces Action Potential Duration at 90% Repolarization and Increases Repolarization Fraction

We next evaluated whether atrial-like expression of marker proteins was associated with typical atrial-like action potential (AP) shape (Figure 5). We used two recently proposed parameters, AP duration (APD) at 90% repolarization (APD_{90}) and repolarization fraction calculated as $(APD_{90} - APD_{50})/APD_{90}$ to distinguish between atrial

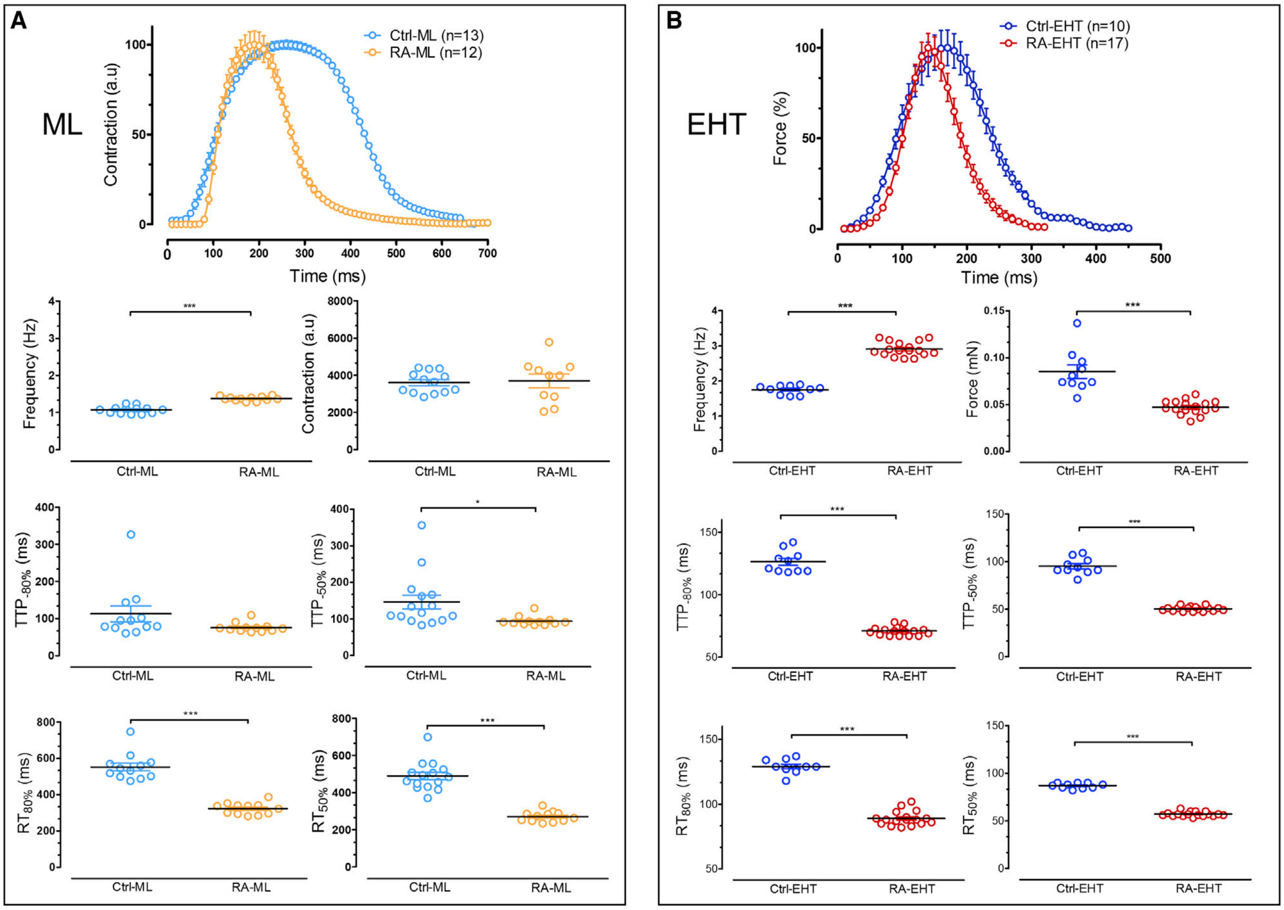

Figure 4. Effect of RA Treatment on Beating Rate and Contraction Kinetics

(A) Effects in MLs: averaged contractions obtained from six different single wells recorded by CellOPTIQ. RA-MLs (n = 13 wells from two batches) showed faster kinetic parameters (TTP and RT) and faster spontaneous beating compared with Ctrl-MLs (n = 12 wells from two batches).

(B) Effects on EHTs: average contraction peaks were calculated from six different EHTs. RA-EHTs (n = 17 from three batches) showed faster contraction kinetics, faster spontaneous beating, and smaller force compared with Ctrl-EHTs (n = 10 from three batches). All average contraction peaks were normalized. Y axes differ between MLs and EHTs to better visualize the effect of RA.

Error bars show means ± SEM.

and ventricular-like hiPSC-CMs (Du et al., 2015). APs were recorded by voltage-sensitive dyes from MLs and by sharp microelectrodes from EHT, RAA, and LV samples. In accordance with a previous report (Lemoine et al., 2018), APD_{90} was shorter in Ctrl-EHTs than in LV. APD_{90} was significantly shorter in RA-MLs and RA-EHTs than in Ctrl-MLs and Ctrl-EHTs, respectively (126 ± 10 ms versus 206 ± 24 ms, n = 70/140 in ML and 166 ± 2 ms versus 243 ± 2 ms, n = 90/157 in EHT; $p < 0.05$, unpaired t test). Repolarization fraction, known to be higher in RAA than in LV and perfectly discriminating between the regions (Horváth et al., 2018), was higher in RA-MLs and RA-EHTs than in Ctrl-MLs and Ctrl-EHTs, respectively (0.28 ± 0.003 versus 0.15 ± 0.002, n = 70/140 in MLs and 0.41 ± 0.005 versus 0.24 ± 0.002, n = 90/157 in EHTs; $p < 0.05$, unpaired t test) with no overlap between the groups (Figures 5 and S7B). These findings indicate a more atrial-like electrophysiological phenotype induced by RA treatment.

RA Treatment Induces Muscarinic Responsiveness of APD

The acetylcholine-activated potassium current $I_{K,ACh}$ is a hallmark of atrial CMs. Its activation by carbachol (CCh) is expected to shorten APD. Based on earlier experiments in human adult cardiac tissues (Dobrev et al., 2001), we expected maximum effects of CCh on AP recordings 2 min after drug exposure. CCh did not have an effect on the APD of LV, but shortened APD_{90} in RAA from 314 ± 14 ms to 174 ± 15 ms (n = 10; $p < 0.05$, paired t test) (Figure 6B). In

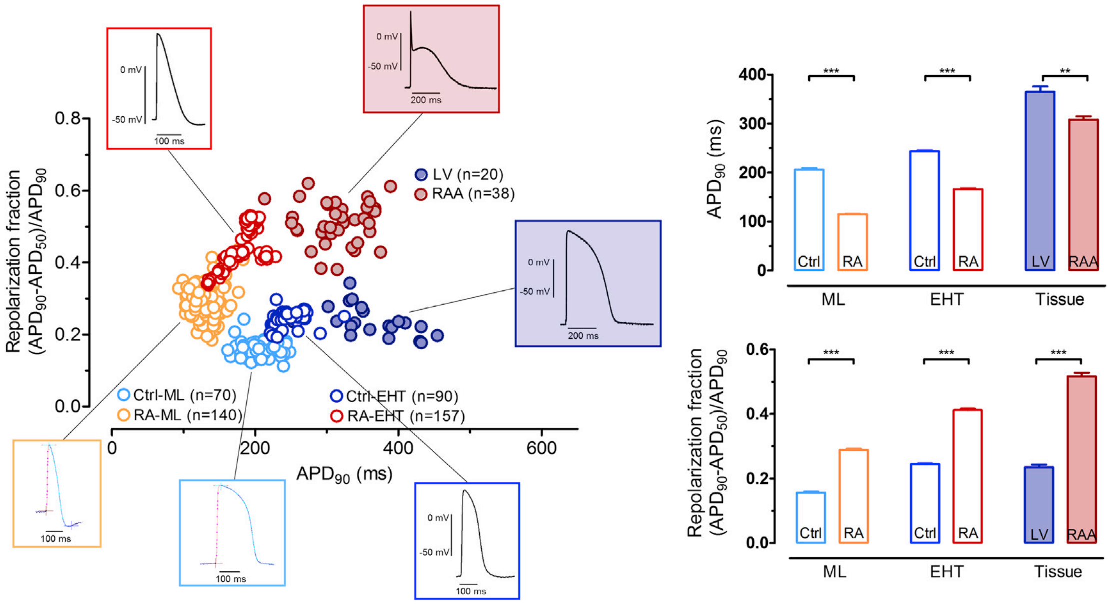

Figure 5. Effect of RA Treatment on APD_{90} and Repolarization Fraction

The scatterplot for APD_{90} versus repolarization fraction includes AP recordings of MLs and EHTs measured by cellOPTIQ and sharp microelectrodes, respectively. For each group a representative original AP trace is shown. The bar graphs (right) show APD_{90} and repolarization fraction $(APD_{90} - APD_{50})/APD_{90}$ in Ctrl-ML (n = 70 wells from two batches), RA-ML (n = 140 wells from two batches), Ctrl-EHT (n = 90/6, number of impalements/EHTs, three batches), RA-EHT (n = 157/6, number of impalements/EHTs, three batches), LV (n = 20 patients), and RAA (n = 38 patients).
Error bars show means ± SEM.

accordance with these data on human heart muscles, CCh reduced APD_{90} of RA-EHT from 221 ± 2.4 to 183 ± 9.4 ms and it did not affect APD_{90} in Ctrl-EHT (Figure 6A). As seen for the resting membrane potential (RMP) in RAA, CCh evoked hyperpolarization of the take-off potential (TOP) in RA-EHTs (from −69.3 ± 3 mV to −73.7 ± 2.8 mV) but not in Ctrl-EHT (Figure 6A).

RA Treatment Makes AP Sensitive to the I_{Kur} Blocker 4-Aminopyridine

In human heart, the ultrarapidly activating delayed rectifier K^+ current (I_{Kur}) is another current predominantly expressed in the atria. We therefore measured the contribution of I_{Kur} to APs using low concentrations of 4-aminopyridine (4-AP; 50 μmol/L) to block I_{Kur}. In RAA, 4-AP prolonged APD_{20} and shortened APD_{90} by 194% and 11.2%, respectively, while it had no effect on LV even at a concentration of 1 mmol/L (Figure 7B) (Wettwer et al., 1994, 2004). In RA-EHTs, 4-AP significantly prolonged APD_{20} (from 31.1 ± 0.14 ms to 44.4 ± 0.82 ms) and AP amplitude (APA), but, in contrast to RAA, did not shorten APD_{90} (Figure S7F). 4-AP had no effect in Ctrl-EHTs (Figure 7A). Prolongation of APD_{20} by 4-AP in RA-EHT but not in Ctrl-EHTs indicates induction of the atrial-selective potassium current I_{Kur} by RA.

DISCUSSION

The aim of this study was to generate a 3D model of human atrial heart muscle. We used recently established RA-based protocols to differentiate atrial-like cells from hiPSCs and used our fibrin-based EHT technique to cast 3D muscle strips that were directly compared with native human heart muscles. The findings show that atrial-like RA-treated hiPSC-CMs readily form spontaneously beating EHTs that, compared with standard EHT, show increased contraction and relaxation velocity, higher repolarization fraction, and pharmacological responses to a muscarinic agonist and an I_{Kur} blocker, characteristic features of atrial heart muscle. The data thus show that addition of RA during hiPSC-CM differentiation not only induces an atrial-like pattern of gene expression but translates into functional properties typically seen in human atrial tissue.

Effect of RA on Atrial-Specific Gene Expression is Larger in EHTs Than in MLs

Several recent studies showed that the presence of RA during hiPSC and hESC differentiation upregulates atrial transcripts such as *COUPTFI*, *COUPTFII*, *PITX2*, *SLN*, *ANP*, and *MLC2A* along with a downregulation of ventricular

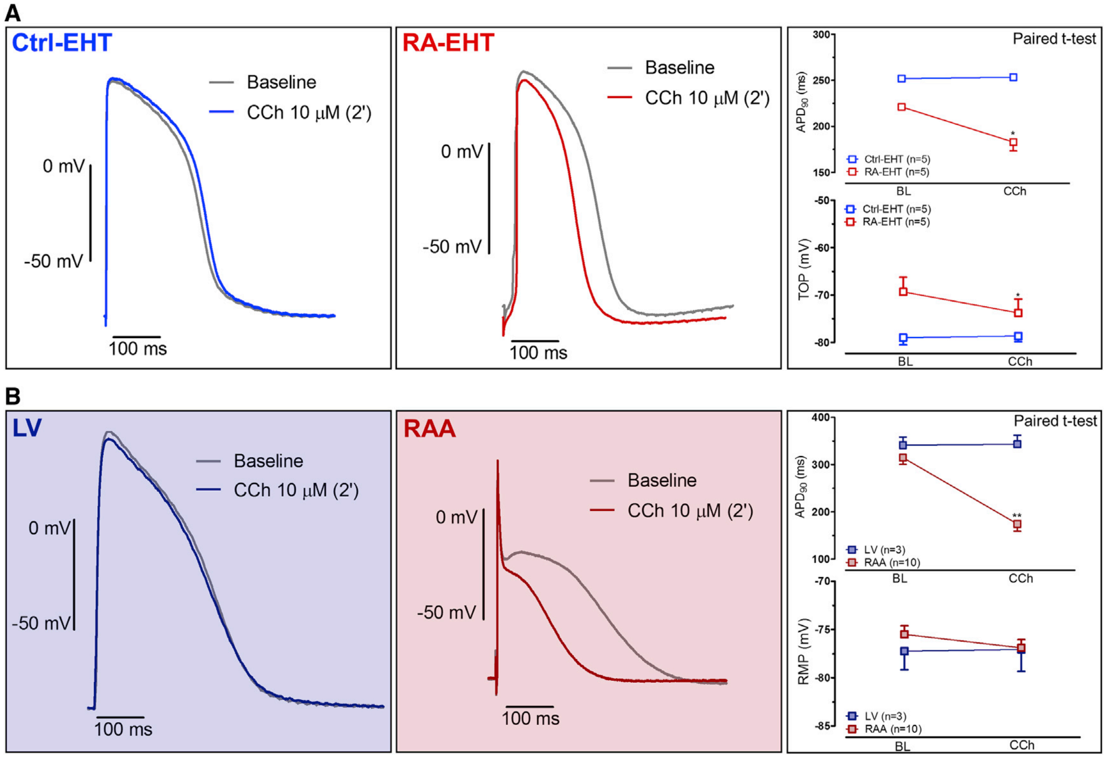

Figure 6. CCh Effect on AP Recordings

Original traces of APs recorded by sharp microelectrodes before and after 2 min exposure to CCh (10 μmol/L) in Ctrl- and RA-EHTs (n = 5 from three batches) (A) and in RAA (n = 10 patients) and LV (n = 3 patients) (B). On the right, mean values for TOP/RMP and APD_{90} are given before and 2 min after CCh exposure. AP traces were recorded at 37°C with 2 Hz pacing for EHTs obtained from ERC18 cell line. LV and RAA APs were field stimulated at 1 Hz. Y axes differ between EHT and human adult cardiac tissues to better visualize the effect of CCh. Error bars show means ± SEM.

transcripts such as *IRX4* and *MLC2V* (Chen et al., 2017; Cyganek et al., 2018; Devalla et al., 2015; Lee et al., 2017; Josowitz et al., 2014; Wu et al., 2013; Zhang et al., 2011). To further characterize the functional phenotype of these cells, we used the EHT technique that improves hiPSC-CM and hESC-CM maturation (Besser et al., 2018; Fong et al., 2016; Mannhardt et al., 2016; Nunes et al., 2013; Ulmer et al., 2018; Uzun et al., 2016; Zhang et al., 2013) and allows direct measurements of force under loaded, steady-state conditions and AP with sharp microelectrodes (Lemoine et al., 2017). An interesting finding was that the differences between Ctrl and RA-treated hiPSC-CMs on gene expression persisted in EHT and were even stronger in EHTs than in MLs in more than 50% of genes (Figure 2). Similarly, the predominance of $MLC2V^+$ cells in Ctrl-treated and of $MLC2A^+$ cells in RA-treated hiPSC-CMs was more pronounced in EHTs than in MLs and approached the almost black-and-white difference in native human ventricular and atrial heart muscles (Figure 3). Thus, the data suggest further specification of chamber-specific characteristics in the 3D EHT format.

RA-EHTs Show Atrial Contraction Pattern

Literature values of contractile force per cross-sectional area for human EHTs range between <0.1 and >20 mN/mm^2. Absolute values, in contrast, range only between 0.08 and 1.5 mN for human EHTs, indicating the relevance of construct diameter for relative force development (Weinberger et al., 2017). In contrast, forces of intact heart muscles can reach 40 to 80 mN/mm^2 (Van Der Velden et al., 1998). The lower forces in EHTs can be mainly explained by lower cardiac myocyte density, lower sarcomere volume fraction, and the overall lower level of cardiac myocyte differentiation (Hirt et al., 2014; Weinberger et al., 2017).

Atrial-specific myosin has a higher cross-bridge cycling rate than ventricular-specific myosin. Since force of a muscle inversely depends on cross-bridge kinetics, tension generation and Ca^{2+} sensitivity of atrial fibers are lower than those of ventricular fibers (Morano et al., 1991). In fact, isometric force of ventricular skinned fibers from adult human heart revealed two times higher force generation per cross section and a higher Ca^{2+} sensitivity than atrial skinned fibers (Ruf et al., 1998; Ng et al., 2010; Piroddi et al., 2007). In

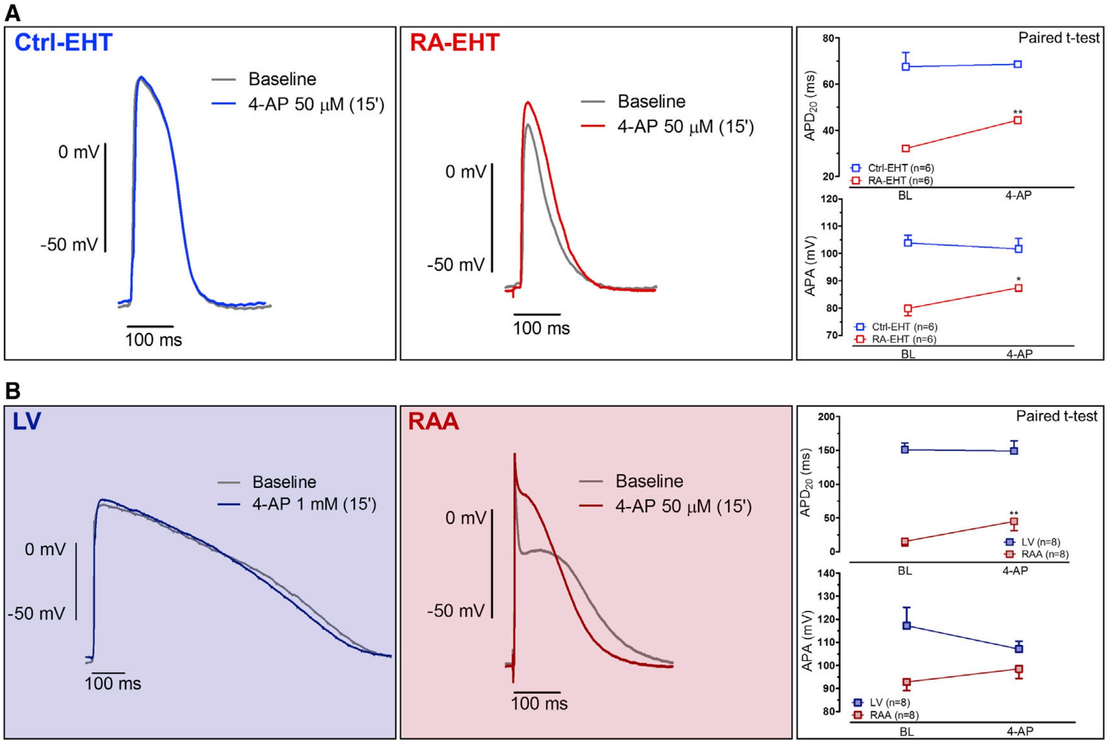

Figure 7. 4-AP Effect on AP Recordings

Original traces of APs recorded by sharp microelectrodes before and after 15 min exposure to 4-AP (50 µmol/L) in Ctrl- and RA-EHTs (n = 6 from three batches) (A) and in RAA (n = 8 patients) and LV (n = 8 patients) (B). On the right, mean values for APD_{20} and APA are given before and 15 min after 4-AP exposure. AP recordings were obtained at 37°C with 2 Hz pacing for Ctrl-EHTs and 4 Hz pacing for RA-EHTs. LV and RAA APs were field stimulated at 1 Hz. Y axes differ between EHT and human adult cardiac tissues to better visualize the effect of 4-AP. Error bars show means ± SEM.

line with this finding, Ctrl-EHTs developed two times higher force than RA-EHTs (Figure 4B).

Furthermore, both the rate of active tension generation and relaxation are faster in atrial than in ventricular myofibrils and this is believed to be related to cross-bridge kinetics (Ng et al., 2010; Piroddi et al., 2007). Atrial and ventricular CMs express predominantly atrial light chain-1 and ventricular light chain-1, respectively. Atrial light chain-1 has faster cross-bridge kinetics than ventricular light chain-1 (Lowey et al., 1993; Morano et al., 1996; Ng et al., 2010). When contracting isometrically, human atrial myocardium exhibited almost two times higher maximum shortening velocity than ventricular myocardium (Berk et al., 2016; Molenaar et al., 2013; Ng et al., 2010). In the present study, RA-EHTs showed 35% shorter $RT_{50\%}$ and 47% shorter $TTP_{-50\%}$ than Ctrl-EHTs, nicely fitting the smaller RT and TTP in human atrial tissue compared with ventricular heart muscle. Qualitatively similar differences were observed by evaluating contractile activity of ML cultured cells by MUSCLEMOTION, but, again, differences between RA and Ctrl were smaller than in EHT (Figure 4). The differences in contractility between RA-EHTs and Ctrl-EHTs may be causally related to the different expression of cardiac myosin light chain isoforms (Figure 3) and to the different sarcomere organization within the EHT (Figure S6A).

RA-EHTs Show Atrial-like AP Parameters

Native atrial and ventricular tissues do not show automaticity. Thus, the spontaneous beating of both RA- and Ctrl-EHTs is a peculiarity of the *in vitro* culture that is well known for all hiPSC-CM models but still not completely understood. Accordingly, the higher beating rate in RA-EHTs compared with Ctrl-EHTs is hard to interpret. RA treatment had a clear impact on AP parameters. RA-EHTs had a less negative TOP than Ctrl-EHTs (−70 ± 1.1 mV versus −76 ± 1.5 mV, Figure S7C), which qualitatively resembles the difference of RMP in human cardiac tissue (−78.5 ± 1.0 mV in LV and −74.0 ± 0.5 mV in RAA) (Burashnikov et al., 2008). The relatively large difference could underlie the lower upstroke velocity in RA-EHTs than in Ctrl-EHTs (97.6 ± 2.4 V/s versus 207.6 ± 10.6 V/s,

Figure S7C), because TOP is in the steep region of the steady-state inactivation curve where small changes in TOP can have large effects on sodium channel availability and subsequently upstroke velocity (Lemoine et al., 2017; Skibsbye et al., 2016).

As seen before, APD_{90} in EHTs from hiPSC-CMs was shorter than in adult human cardiac tissue (Horváth et al., 2018). Nevertheless, RA treatment of hiPSC-CMs further shortened APD_{90} (Figure 5). The difference between RA and Ctrl was larger than the difference between RAA and LV. Since refractoriness depends strictly on membrane voltage and therefore on APD_{90}, the short AP in RA-EHTs could facilitate tachyarrhythmias, which may come as an advantage for future drug testing purposes.

Repolarization fraction is known to better discriminate atrial versus ventricular APs than the absolute values of APD_{90} (Du et al., 2015; Horváth et al., 2018). We observed the same in the present study. As seen in RAA versus LV before (Horváth et al., 2018), repolarization fraction did not overlap between RA-EHT and Ctrl-EHT, suggesting a strong effect of RA to induce an atrial-like repolarization pattern. However, we never saw the very rapid initial repolarization leading to a pronounced spike and dome phenomenon typical for human RAA. The shape of RA-EHT APs resembled APs recorded from patients with persistent AF and a loss of the steep initial repolarization. This finding could suggest a low contribution of transient potassium outward currents as I_{to} and I_{Kur}.

RA-EHTs Respond to Atrial-Selective Drugs

Ion channels Kv1.5 and Kir3.1, encoded by KCNA5 and KCNJ3, respectively, conduct the potassium currents I_{Kur} and $I_{K,ACh}$, which are major determinants of electrophysiological differences between atrial and ventricular CMs (Ravens et al., 2013). Here we have used CCh to identify $I_{K,ACh}$. Both RA-EHTs and Ctrl-EHTs decreased beating rate upon CCh exposure (Figure S7D). However, this finding cannot be taken as a proof for $I_{K,ACh}$, since activation of muscarinic receptors also effectively decreases I_f (DiFrancesco et al., 1989). On the other hand, APD shortening upon muscarinic receptor activation should allow clear $I_{K,Ach}$ identification. The absence of CCh-induced APD shortening in Ctrl-EHTs is in line with the absence of $I_{K,ACh}$ in cells isolated from Ctrl-EHTs (Horváth et al., 2018). In contrast, CCh-induced APD shortening in RA-EHTs is an indication of an atrial-like phenotype. The effect of CCh was tested on Ctrl- and RA-EHTs obtained from another Ctrl cell line, ERC18. This cell line showed lower baseline beating frequency than C25 (2 Hz versus 3 Hz). Given that the effect of CCh on APD_{90} shows a reversed rate-dependency (i.e., decreases at higher frequency) (Figure S7E), we expected a larger effect in ERC18 (Figure S3). Indeed, CCh shortened APD_{90} in RA-EHTs by 20% in ERC18 compared with 7% in C25. The effect was still smaller than the 50% shortening in RAA (Figure 6), likely due to the lower current amplitude of $I_{K,Ach}$ in RA-EHTs (Figure 6).

I_{Kur} is another atrial-selective potassium current recently employed to identify atrial-like repolarization pattern. The recently developed selective I_{Kur} blocker Xention D-0101 was effective in RA-treated but not in Ctrl hESC (Devalla et al., 2015; Ford et al., 2013). Since this compound was not available, we used 4-AP at low concentration (50 μmol/L). Even when we used high concentrations (1 mmol/L) of 4-AP, we could not detect an effect in LV. This finding may be due to the use of subendocardial preparations from patients with heart failure showing very low I_{to} amplitudes (Wettwer et al., 1994). At 50 μmol/L, 4-AP resembles the effects of selective I_{Kur} block by D-0101 in RAA; that is, prolongation of APD_{20} but shortening of APD_{90} (Ford et al., 2013; Wettwer et al., 2004). We confirmed this effect in RAA (Figure 7B). In RA-EHTs, 4-AP (50 μmol/L) induced the expected prolongation of APD_{20} but did not shorten APD_{90}. The shortening of APD_{90} by low concentration of 4-AP in RAA results from stronger activation of I_{Kr} by a more positive plateau voltage induced by the prolongation of APD_{20} (Wettwer et al., 2004). Maybe the absence of a clear plateau phase in RA-EHTs limits indirect effects of I_{Kur} block on I_{Kr}.

Taken together, the present results confirm and extend previous findings demonstrating that RA induces atrial CM specification during cardiac differentiation from hiPSCs and hESCs. The 3D EHT format accentuated atrial versus ventricular differences and revealed characteristic atrial heart muscle features in terms of gene expression, contractile force, contraction kinetics, AP features, and pharmacological responses. While quantitative differences to native human atrial myocardium remain, the data overall suggest that RA-EHTs may be a useful extension of experimental models in preclinical drug development and mechanistic studies. Particularly relevant would be chronic pacing of the RA-EHTs to study electrical remodeling of AF and test new potential drugs.

EXPERIMENTAL PROCEDURES

Differentiation of hiPSC-CMs

This investigation conforms to the principles outlined by the Declaration of Helsinki and the Medical Association of Hamburg. All materials from patients were taken with informed consent of the donors. All procedures involving the generation and analysis of hiPSC lines were approved by the local ethics committee in Hamburg (Az PV4798, 28.10.2014).

Expansion of three undifferentiated hiPSC Ctrl cell lines (C25, ERC18, and ERC1) (Figure S3) was performed in FTDA medium, as recently described (Breckwoldt et al., 2017). Embryoid body (EB) formation was induced in stirred suspension cultures

(spinner flasks). Mesodermal induction was achieved using BMP-4 (10 ng/mL), activin A (3 ng/mL), and bFGF (5 ng/mL) in the absence of insulin in RPMI medium (Breckwoldt et al., 2017). Specification of cardiac differentiation of mesodermal progenitors was performed by WNT signal inhibition (XAV939, 1 μmol/L). This resulted in a population of primarily ventricular CMs. Based on previous reports (Cyganek et al., 2018; Devalla et al., 2015; Lee et al., 2017; Zhang et al., 2011), differentiation of atrial-like myocytes was achieved by RA treatment (1 μmol/L) for the first 3 days of Wnt signaling inhibition. RA (Sigma Aldrich R2625) was prepared as explained in a previous publication (Devalla et al., 2015). Data presented in this manuscript were mainly derived from the Ctrl cell line C25 (Moretti et al., 2010) but are representative of similar results in all three lines (Figure S3). All data were confirmed in at least three batches.

ML and EHT Generation

At the end of cardiac differentiation, no purification step was performed before ML and EHT preparation. Differentiated EBs were enzymatically dispersed with collagenase II (200 U/mL, Worthington, LS004176 in Hank's balanced salt solution minus Ca^{2+}/Mg^{2+}, Gibco, 14175-053) for 3.5 hr at 37°C (Breckwoldt et al., 2017). Part of the dissociated cells were plated onto black-sided 96-well plates (NUNC; 10,000 cells/well) pre-coated with Geltrex (Gibco, A1413302; 1:100 in DMEM, 37°C, 1 hr). After 14 days in culture, the cells formed a uniform ML. At the same time, dissociated cells were mixed with fibrinogen (Sigma F4753) and thrombin (100 U/mL, Sigma Aldrich T7513) to cast EHTs (1×10^6 cells/EHT) (Breckwoldt et al., 2017). The EHT is a synchronously beating syncytium of hiPSC-CMs that generates auxotonic contractile force by deflecting two elastic silicone posts (Breckwoldt et al., 2017; Hansen et al., 2010; Mannhardt et al., 2016). After 14 days in culture, EHTs displayed spontaneous coherent, regular beating deflecting the silicone posts and allowed video-optical contraction analysis.

Contractility Measurements

MLs

Contraction activity in MLs was measured using an established platform (CellOPTIQ, Clyde Biosciences, UK). In brief, ventricular- and atrial-like myocytes obtained from hiPSCs were seeded onto 96-well glass-bottomed plates (MatTek, p96G-1.5-5-F) pre-coated with 1:100 fibronectin (Sigma, F1141) in Dulbecco's phosphate-buffered saline (Gibco, ThermoFisher Scientific, UK, 14040133) for 3 hr at 37°C before plating. A total volume of 200 μL of cell suspension was used to obtain a final density of 65,000 cells/cm^2. After 14 days, videos of MLs were acquired at a sampling rate of 100 frames/s for 10 s. During recordings, the 96-well plate was positioned inside an on-stage incubator at 37°C with 5% CO_2. Camera: Hamamatsu ORCA-flash4.0 V2 digital CMOS camera C13440–20CU. Microscope: Olympus IX73. Objective: Olympus, 40× air, numerical aperture 0.60. Spontaneous contraction activity of cells was measured using the algorithm of MUSCLEMOTION software (Sala et al., 2018). Average contraction peaks were generated from six contraction peaks of different MLs. These average peaks were normalized to the smallest and largest values in the dataset and depicted as mean ± SEM.

EHTs

Contractile analysis was performed on 14- to 20-day-old EHTs in modified Tyrode's solution (in mmol/L: NaCl, 120; KCl, 5.4; $MgCl_2$, 1; $CaCl_2$, 1.8; NaH_2PO_4, 0.4; $NaHCO_3$, 22.6; glucose, 5; Na_2EDTA, 0.05; and HEPES, 25) pre-equilibrated overnight (37°C, 7% CO_2, 40% O_2). Analysis of contractile force was performed by video-optical recording as previously described (Hansen et al., 2010; Schaaf et al., 2011) on a setup available from EHT Technologies. The contraction peaks were analyzed in terms of frequency, force, TTP, and RT. $TTP_{-80\%}$ and $TTP_{-50\%}$ refer to the time from 20% to 50% above baseline (−80% and −50% from peak) to peak, respectively. $RT_{50\%}$ and $RT_{80\%}$ refer to the time from peak to 50% and 80% relaxation (50% and 20% above baseline), respectively. Average contraction peaks were generated from six contraction peaks of different EHTs. These average peaks were normalized to the smallest and largest values in the dataset and depicted as mean ± SEM.

AP Recordings

MLs

APs in MLs were measured using CellOPTIQ platform, as previously described for ML contractility measurements. After 2 weeks, cells were transferred to serum-free media (DMEM, Gibco 11966, supplemented with galactose 10 mM and sodium pyruvate 1 mM). Cells were exposed for 1 min to the ratiometric voltage-sensitive dye (Di-4-ANEPPS 6 μmol/L, at room temperature). Afterward, MLs were placed on a heated platform of an inverted microscope (37°C and 95% O_2, 5% CO_2). The Di-4-ANEPPS fluorescence was recorded at 10 kHz for 15 s in each well. Voltage signals were subsequently analyzed offline using proprietary software (Clyde Biosciences).

EHTs

APs in EHTs were recorded with sharp microelectrodes (14–20 days old) as previously described (Lemoine et al., 2017, 2018). Tissue from human LVs and RAAs was used for comparison. Tissues were continuously superfused with Tyrode's solution (NaCl, 127 mmol/L; KCl, 5.4 mmol/L; $MgCl_2$, 1.05 mmol/L; $CaCl_2$, 1.8 mmol/L; glucose, 10 mmol/L; $NaHCO_3$, 22 mmol/L; $NaHPO_4$, 0.42 mmol/L; equilibrated with O_2-CO_2 [95:5] at 36.5°C ± 0.5°C, pH 7.4). Tissues were field stimulated with a rectangular pulse of 1 ms at a fixed rate 50% above threshold. All parameters related to APD were corrected for the beating rate with Bazett correction (Bazett, 1997). RAA samples were obtained from patients suffering from coronary artery disease or valve disease undergoing bypass or valve replacement. Left ventricular free wall samples were obtained from patients suffering from valve disease or cardiomyopathy (details are given in Table S2). All patients gave informed consent. The study followed the Declaration of Helsinki. Drug effects were measured 2 min after exposure to CCh and 15 min after exposure to 4-AP. APs were analyzed offline using the Lab-Chart software (ADInstruments, Spechbach, Germany).

Statistics

Statistical analyses were performed with GraphPad Prism software 5.0. Data are expressed as mean ± SEM in bar graphs and scatterplots. Differences between groups were analyzed by paired or unpaired t test. Results were considered statistically significant if

the p value was less than 0.05. All experiments consisted of at least three independent batches.

SUPPLEMENTAL INFORMATION

Supplemental Information includes Supplemental Experimental Procedures, seven figures, two tables, and two videos and can be found with this article online at https://doi.org/10.1016/j.stemcr.2018.10.008.

AUTHOR CONTRIBUTIONS

M.L., B.M.U., M.D.L., G.S., A.H., T.C., and T.E. planned experiments. M.L., B.M.U., M.D.L., A.T.L.Z., F.F., U.R., H.R., and M.R.-G. contributed to experiments and data analysis. M.L., T.C., and T.E. wrote the manuscript. All authors approved the final version of the manuscript.

ACKNOWLEDGMENTS

The authors would like to thank Professor Robert Passier for the helpful visit at Leiden University Medical Center to learn their expertise to differentiate atrial-like myocytes from hESCs. Authors thank the members of the hiPSC-CM working group at the Department of Experimental Pharmacology and Toxicology, UKE-Hamburg, for their support with stem cell culture and CM differentiation. We would like to thank PD Dr. Monica Patten, MD (University Heart Center, UKE) and Prof. Lucie Carrier (Department of Experimental Pharmacology and Toxicology, UKE) for providing access to myectomy samples from patients with hypertrophic cardiomyopathy. We are very grateful to Mirja L. Schulze, Aya Shibamiya and Elisabeth Schulze (UKE) for providing the ERC18 and ERC1 Ctrl hiPSC lines. The C25 Ctrl hiPSC line was provided by the group of Alessandra Moretti, Munich. The FACS analysis was done with support from the FACS Core unit of the UKE. We greatly appreciate the assistance of Susanne Krasemann and Kristin Hartmann (mouse pathology core facility of the UKE) in immunohistochemistry and TEM imaging. The work was supported by the European Union's Horizon 2020 research and innovation programme under the Marie Skłodowska-Curie grant agreement no. 675351, the German Ministry of Education and Research (BMBF) and German Centre for Cardiovascular Research (DZHK 81Z0710101), the European Research Council (ERC AG IndivuHeart), the Deutsche Forschungsgemeinschaft (DFG, HA 3423-5-1), and Leducq Fondation (research grant Nr. 11, CVD04). T.E. and A.H. are co-founders of EHT Technologies, Hamburg. G.S. is a founder and executive of Clyde Biosciences Ltd (UK).

Received: May 15, 2018
Revised: October 9, 2018
Accepted: October 10, 2018
Published: November 8, 2018

REFERENCES

Bazett, H.C. (1997). An analysis of the time-relations of electrocardiograms. Ann. Noninvasive Electrocardiol. *2*, 177–194.

Bensley, J.G., De Matteo, R., Harding, R., and Black, M.J. (2016). Three-dimensional direct measurement of cardiomyocyte volume, nuclearity, and ploidy in thick histological sections. Sci. Rep. *6*, 1–10.

Berk, E., Christ, T., Schwarz, S., Ravens, U., Knaut, M., and Kaumann, A.J. (2016). In permanent atrial fibrillation, PDE3 reduces force responses to 5-HT, but PDE3 and PDE4 do not cause the blunting of atrial arrhythmias. Br. J. Pharmacol. *173*, 2478–2489.

Besser, R.R., Ishahak, M., Mayo, V., Carbonero, D., Claure, I., and Agarwal, A. (2018). Engineered microenvironments for maturation of stem cell derived cardiac myocytes. Theranostics *8*, 124–140.

Blazeski, A., Renjun, Z., Hunter, D.W., Weinberg, S.H., Zambidis, E.T., and Tung, L. (2012). Cardiomyocytes derived from human induced pluripotent stem cells as models for normal and diseased cardiac electrophysiology and contractility. Prog. Biophys. Mol. Biol. *110*, 166–177.

Breckwoldt, K., Letuffe-Brenière, D., Mannhardt, I., Schulze, T., Ulmer, B., Werner, T., Benzin, A., Klampe, B., Reinsch, M.C., Laufer, S., et al. (2017). Differentiation of cardiomyocytes and generation of human engineered heart tissue. Nat. Protoc. *12*, 1177–1197.

Burashnikov, A., di Diego, J.M., Zygmunt, A.C., Belardinelli, L., and Antzelevitch, C. (2008). Atrial-selective sodium channel block as a strategy for suppression of atrial fibrillation. Ann. N. Y. Acad. Sci. *1123*, 105–112.

Chen, Z., Xian, W., Bellin, M., Dorn, T., Tian, Q., Goedel, A., Dreizehnter, L., Schneider, C.M., Ward-van Oostwaard, D., Ng, J.K., et al. (2017). Subtype-specific promoter-driven action potential imaging for precise disease modelling and drug testing in hiPSC-derived cardiomyocytes. Eur. Heart J. *38*, 292–301.

Chugh, S.S., Havmoeller, R., Narayanan, K., Singh, D., Rienstra, M., Benjamin, E.J., Gillum, R.F., Kim, Y.H., McAnulty, J.H., Jr., and Zheng, Z.J. (2014). Worldwide epidemiology of atrial fibrillation: a global burden of disease 2010 study. Circulation *129*, 837–847.

Claycomb, W.C., Delcarpio, J.B., Guice, S.E., and Moses, R.L. (1989). Culture and characterization of fetal human atrial and ventricular cardiac muscle cells. In Vitro Cell. Dev. Biol. *25*, 1114–1120.

Cyganek, L., Tiburcy, M., Sekeres, K., Gerstenberg, K., Bohnenberger, H., Lenz, C., Henze, S., Stauske, M., Salinas, G., Zimmermann, W.-H., et al. (2018). Deep phenotyping of human induced pluripotent stem cell–derived atrial and ventricular cardiomyocytes. JCI Insight *3*. https://doi.org/10.1172/jci.insight.99941.

Denayer, T., Stöhr, T., and Van Roy, M. (2014). Animal models in translational medicine: validation and prediction. New Horiz. Transl. Med. *2*, 5–11.

Devalla, H.D., Schwach, V., Ford, J.W., Milnes, J.T., El-Haou, S., Jackson, C., Gkatzis, K., Elliott, D.A., Chuva de Sousa Lopes, S.M., Mummery, C.L., et al. (2015). Atrial-like cardiomyocytes from human pluripotent stem cells are a robust preclinical model for assessing atrial-selective pharmacology. EMBO Mol. Med. *7*, 394–410.

DiFrancesco, D., Ducouret, P., and Robinson, R.B. (1989). Muscarinic modulation of cardiac rate at low acetylcholine concentrations. Science *243*, 669–671.

Dobrev, D., Graf, E., Wettwer, E., Himmel, H.M., Hála, O., Doerfel, C., Christ, T., Schüler, S., and Ravens, U. (2001). Molecular basis of downregulation of G-protein-coupled inward rectifying K(+)

current (I(K,ACh) in chronic human atrial fibrillation: decrease in GIRK4 mRNA correlates with reduced I(K,ACh) and muscarinic receptor-mediated shortening of action potentials. Circulation *104*, 2551–2557.

Du, D.T.M., Hellen, N., Kane, C., and Terracciano, C.M.N. (2015). Action potential morphology of human induced pluripotent stem cell-derived cardiomyocytes does not predict cardiac chamber specificity and is dependent on cell density. Biophys. J. *108*, 1–4.

Ellinghaus, P., Scheubel, R.J., Dobrev, D., Ravens, U., Holtz, J., Huetter, J., Nielsch, U., and Morawietz, H. (2005). Comparing the global mRNA expression profile of human atrial and ventricular myocardium with high-density oligonucleotide arrays. J. Thorac. Cardiovasc. Surg. *129*, 1383–1390.

Fong, A.H., Romero-López, M., Heylman, C.M., Keating, M., Tran, D., Sobrino, A., Tran, A.Q., Pham, H.H., Fimbres, C., Gershon, P.D., et al. (2016). Three-dimensional adult cardiac extracellular matrix promotes maturation of human induced pluripotent stem cell-derived cardiomyocytes. Tissue Eng. Part A *22*, 1016–1025.

Ford, J., Milnes, J., Wettwer, E., Christ, T., Rogers, M., Sutton, K., Madge, D., Virag, L., Jost, N., Horvath, Z., et al. (2013). Human electrophysiological and pharmacological properties of XEN-D0101: a novel atrial-selective Kv1.5/IKur inhibitor. J. Cardiovasc. Pharmacol. *61*, 408–415.

Gaborit, N., Le Bouter, S., Szuts, V., Varro, A., Escande, D., Nattel, S., and Demolombe, S. (2007). Regional and tissue specific transcript signatures of ion channel genes in the non-diseased human heart. J. Physiol. *582*, 675–693.

Hansen, A., Eder, A., Bönstrup, M., Flato, M., Mewe, M., Schaaf, S., Aksehirlioglu, B., Schwörer, A., Uebeler, J., and Eschenhagen, T. (2010). Development of a drug screening platform based on engineered heart tissue. Circ. Res. *107*, 35–44.

Hirt, M.N., Boeddinghaus, J., Mitchell, A., Schaaf, S., Börnchen, C., Müller, C., Schulz, H., Hubner, N., Stenzig, J., Stoehr, A., et al. (2014). Functional improvement and maturation of rat and human engineered heart tissue by chronic electrical stimulation. J. Mol. Cell. Cardiol. *74*, 151–161.

Horváth, A., Lemoine, M.D., Löser, A., Mannhardt, I., Flenner, F., Uzun, A.U., Neuber, C., Breckwoldt, K., Hansen, A., Girdauskas, E., et al. (2018). Low resting membrane potential and low inward rectifier potassium currents are not inherent features of hiPSC-derived cardiomyocytes. Stem Cell Reports *10*, 822–833.

Josowitz, R., Lu, J., Falce, C., D'Souza, S.L., Wu, M., Cohen, N., Dubois, N.C., Zhao, Y., Sobie, E.A., Fishman, G.I., et al. (2014). Identification and purification of human induced pluripotent stem cell-derived atrial-like cardiomyocytes based on sarcolipin expression. PLoS One *9*, e101316.

Lee, J.H., Protze, S.I., Laksman, Z., Backx, P.H., and Keller, G.M. (2017). Human pluripotent stem cell-derived atrial and ventricular cardiomyocytes develop from distinct mesoderm populations. Cell Stem Cell *21*, 179–194.e4.

Lemoine, M.D., Mannhardt, I., Breckwoldt, K., Prondzynski, M., Flenner, F., Ulmer, B., Hirt, M.N., Neuber, C., Horváth, A., Kloth, B., et al. (2017). Human iPSC-derived cardiomyocytes cultured in 3D engineered heart tissue show physiological upstroke velocity and sodium current density. Sci. Rep. *7*, 1–11.

Lemoine, M.D., Krause, T., Koivumäki, J.T., Prondzynski, M., Schulze, M.L., Girdauskas, E., Willems, S., Hansen, A., Eschenhagen, T., and Christ, T. (2018). Human induced pluripotent stem cell–derived engineered heart tissue as a sensitive test system for QT prolongation and arrhythmic triggers. Circ. Arrhythm. Electrophysiol. *11*, e006035.

Lowey, S., Waller, S.G., and Trybus, M.K. (1993). Skeletal muscle myosin light chains are essential for physiological speeds of shortening. Nature *366*, 461–464.

Ma, J., Guo, L., Fiene, S.J., Anson, B.D., Thomson, J.A., Kamp, T.J., Kolaja, K.L., Swanson, B.J., January, C.T., Kl, K., et al. (2011). High purity human-induced pluripotent stem cell-derived cardiomyocytes: electrophysiological properties of action potentials and ionic currents. Am. J. Physiol. Heart Circ. Physiol. *301*, 2006–2017.

Mannhardt, I., Breckwoldt, K., Letuffe-Brenière, D., Schaaf, S., Schulz, H., Neuber, C., Benzin, A., Werner, T., Eder, A., Schulze, T., et al. (2016). Human engineered heart tissue: analysis of contractile force. Stem Cell Reports *7*, 29–42.

Marczenke, M., Piccini, I., Mengarelli, I., Fell, J., Röpke, A., Seebohm, G., Verkerk, A.O., and Greber, B. (2017). Cardiac subtype-specific modeling of Kv1.5 ion channel deficiency using human pluripotent stem cells. Front. Physiol. *8*, 1–11.

Marini, C., De Santis, F., Sacco, S., Russo, T., Olivieri, L., Totaro, R., and Carolei, A. (2005). Contribution of atrial fibrillation to incidence and outcome of ischemic stroke: results from a population-based study. Stroke *36*, 1115–1119.

Molenaar, P., Christ, T., Hussain, R.I., Engel, A., Berk, E., Gillette, K.T., Chen, L., Galindo-Tovar, A., Krobert, K.A., Ravens, U., et al. (2013). PDE3, but not PDE4, reduces β1- and β2-adrenoceptor-mediated inotropic and lusitropic effects in failing ventricle from metoprolol-treated patients. Br. J. Pharmacol. *169*, 528–538.

Morano, I., Bletz, C., Wojciechowski, R., and Rüegg, J. (1991). Brief communication modulation of crossbridge kinetics by myosin isoenzymes in skinned human heart fibers. Circ. Res. *68*, 614–619.

Morano, M., Zacharzowski, U., Maier, M., Lange, P.E., Alexi-Meskishvili, V., Haase, H., and Morano, I. (1996). Regulation of human heart contractility by essential myosin light chain isoforms. J. Clin. Invest. *98*, 467–473.

Moretti, A., Bellin, M., Welling, A., and Billy Jung, C. (2010). Patient-specific induced pluripotent stem-cell models for long-QT syndrome. N. Engl. J. Med. *363*, 1397–1409.

Mosqueira, D., Mannhardt, I., Bhagwan, J.R., Lis-Slimak, K., Katili, P., Scott, E., Hassan, M., Prondzynski, M., Harmer, S.C., Tinker, A., et al. (2018). CRISPR/Cas9 editing in human pluripotent stem cell-cardiomyocytes highlights arrhythmias, hypocontractility, and energy depletion as potential therapeutic targets for hypertrophic cardiomyopathy. Eur. Heart J., 1–16. https://doi.org/10.1093/eurheartj/ehy249.

Ng, S.Y., Wong, C.K., and Tsang, S.Y. (2010). Differential gene expressions in atrial and ventricular myocytes: insights into the road of applying embryonic stem cell-derived cardiomyocytes for future therapies. Am. J. Physiol. Physiol. *299*, C1234–C1249.

Nunes, S.S., Miklas, J.W., Liu, J., Aschar-sobbi, R., Xiao, Y., Zhang, B., Jiang, J., Masse, S., Gagliardi, M., Hsieh, A., et al. (2013). Biowire: a new platform for maturation of human pluripotent stem cell derived cardiomyocytes. Nat. Methods *10*, 781–787.

Piroddi, N., Belus, A., Scellini, B., Tesi, C., Giunti, G., Cerbai, E., Mugelli, A., and Poggesi, C. (2007). Tension generation and relaxation in single myofibrils from human atrial and ventricular myocardium. Pflugers Arch. Eur. J. Physiol. *454*, 63–73.

Ravens, U., Poulet, C., Wettwer, E., and Knaut, M. (2013). Atrial selectivity of antiarrhythmic drugs. J. Physiol. *591*, 4087–4097.

Ruf, T., Schulte-Baukloh, H., Lüdemann, J., Posival, H., Beyersdorf, F., Just, H., and Holubarsh, C. (1998). Alterations of cross-bridge kinetics in human atrial and ventricular myocardium. Cardiovasc. Res. *40*, 580–590.

Sala, L., van Meer, B.J., Tertoolen, L.T., Bakkers, J., Bellin, M., Davis, R.P., Denning, C.N., Dieben, M.A., Eschenhagen, T., Giacomelli, E., et al. (2018). MUSCLEMOTION: a versatile open software tool to quantify cardiomyocyte and cardiac muscle contraction in vitro and in vivo. Circ. Res. *122*, e5–e16.

Schaaf, S., Shibamiya, A., Mewe, M., Eder, A., Stöhr, A., Hirt, M.N., Rau, T., Zimmermann, W.H., Conradi, L., Eschenhagen, T., et al. (2011). Human engineered heart tissue as a versatile tool in basic research and preclinical toxicology. PLoS One *6*, e26397.

Schotten, U., Verheule, S., Kirchhof, P., and Goette, A. (2011). Pathophysiological mechanisms of atrial fibrillation: a translational appraisal. Physiol. Rev. *91*, 265–325.

Skibsbye, L., Jespersen, T., Christ, T., Maleckar, M.M., van den Brink, J., Tavi, P., and Koivumäki, J.T. (2016). Refractoriness in human atria: time and voltage dependence of sodium channel availability. J. Mol. Cell. Cardiol. *101*, 26–34.

Ulmer, B.M., Stoehr, A., Schulze, M.L., Patel, S., Gucek, M., Mannhardt, I., Funcke, S., Murphy, E., Eschenhagen, T., and Hansen, A. (2018). Contractile work contributes to maturation of energy metabolism in hiPSC-derived cardiomyocytes. Stem Cell Reports *10*, 834–847.

Uzun, A.U., Mannhardt, I., Breckwoldt, K., Horváth, A., Johannsen, S.S., Hansen, A., Eschenhagen, T., and Christ, T. (2016). Ca2+-currents in human induced pluripotent stem cell-derived cardiomyocytes effects of two different culture conditions. Front. Pharmacol. *7*, 1–14.

van den Berg, C.W., Elliott, D.A., Braam, S.R., Mummery, C.L., and Davis, R.P. (2016). Differentiation of human pluripotent stem cells to cardiomyocytes under defined conditions. Methods Mol. Biol. *1341*, 257–284.

Van Der Velden, J., Klein, L.J., Van Der Bijl, M., Huybregts, M.A.J.M., Stooker, W., Witkop, J., Eijsman, L., Visser, C.A., Visser, F.C., and Stienen, G.J.M. (1998). Force production in mechanically isolated cardiac myocytes from human ventricular muscle tissue. Cardiovasc. Res. *38*, 414–423.

Vollert, I., Seiffert, M., Bachmair, J., Sander, M., Eder, A., Conradi, L., Vogelsang, A., Schulze, T., Uebeler, J., Holnthoner, W., et al. (2013). In-vitro perfusion of engineered heart tissue through endothelialized channels. Tissue Eng. Part A *20*, 854–863.

Wang, T.J., Massaro, J.M., Levy, D., Vasan, R.S., Wolf, P.A., D'Agostino, R.B., Larson, M.G., Kannel, W.B., and Benjamin, E.J. (2003). A risk score for predicting stroke or death in individuals with new-onset atrial fibrillation in the community. JAMA *290*, 1049–1056.

Weinberger, F., Mannhardt, I., and Eschenhagen, T. (2017). Engineering cardiac muscle tissue: a maturating field of research. Circ. Res. *120*, 1487–1500.

Wettwer, E., Amos, G.J., Posival, H., and Ravens, U. (1994). Transient outward current in human ventricular myocytes of subepicardial and subendocardial origin. Circ. Res. *75*, 473–482.

Wettwer, E., Hála, O., Christ, T., Heubach, J.F., Dobrev, D., Knaut, M., Varró, A., and Ravens, U. (2004). Role of IKur in controlling action potential shape and contractility in the human atrium: influence of chronic atrial fibrillation. Circulation *110*, 2299–2306.

Wobus, A.M., Rohwedel, J., Maltsev, V., and Hescheler, J. (1995). Development of cardiomyocytes expressing cardiac-specific genes, action potentials, and ionic channels during embryonic stem cell-derived cardiogenesis. Ann. N. Y. Acad. Sci. *752*, 460–469.

Wu, S.P., Cheng, C.M., Lanz, R.B., Wang, T., Respress, J.L., Ather, S., Chen, W., Tsai, S.J., Wehrens, X.H.T., Tsai, M.J., et al. (2013). Atrial identity is determined by a COUP-TFII regulatory network. Dev. Cell *25*, 417–426.

Zaffran, S., Robrini, N., and Bertrand, N. (2014). Retinoids and cardiac development. J. Dev. Biol. *2*, 50–71.

Zhang, Q., Jiang, J., Han, P., Yuan, Q., Zhang, J., Zhang, X., Xu, Y., Cao, H., Meng, Q., Chen, L., et al. (2011). Direct differentiation of atrial and ventricular myocytes from human embryonic stem cells by alternating retinoid signals. Cell Res. *21*, 579–587.

Zhang, D., Shadrin, I., Lam, J., Xian, H.-Q., Snodgrass, R., and Bursac, N. (2013). Tissue-engineered cardiac patch for advanced functional maturation of human ESC-derived cardiomyocytes. Biomaterials *34*, 5813–5820.

Stem Cell Reports, Volume *11*

Supplemental Information

Atrial-like Engineered Heart Tissue: An *In Vitro* Model of the Human Atrium

Marta Lemme, Bärbel M. Ulmer, Marc D. Lemoine, Antonia T.L. Zech, Frederik Flenner, Ursula Ravens, Hermann Reichenspurner, Miriam Rol-Garcia, Godfrey Smith, Arne Hansen, Torsten Christ, and Thomas Eschenhagen

Supplemental Information

Supplemental Data

Figure S1. Negative controls for flow cytometry, related to Figure 1B

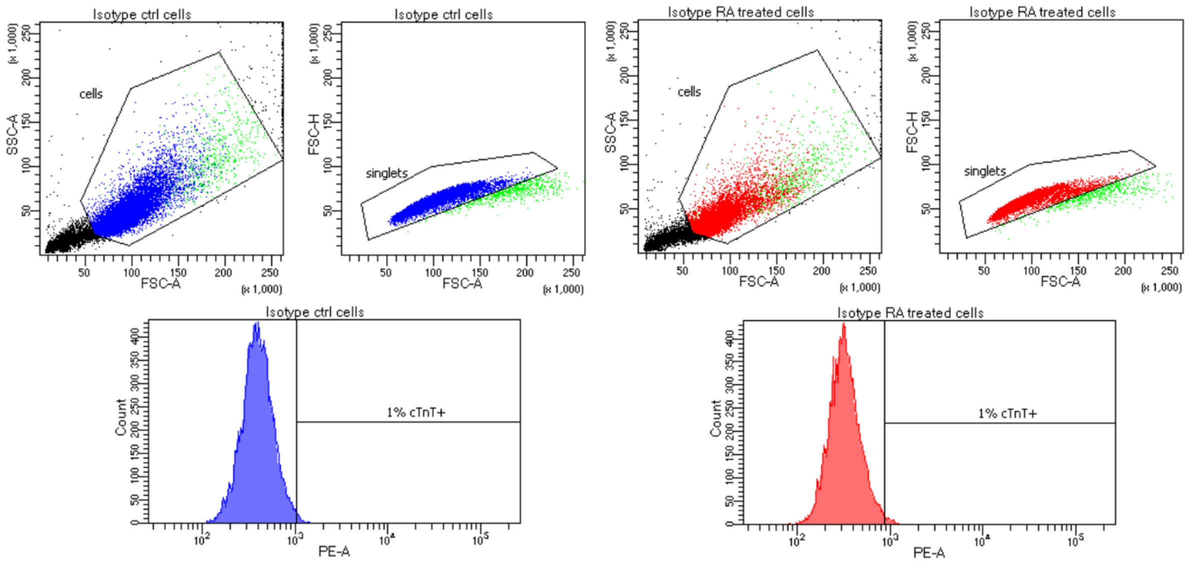

Figure S1. Flow cytometry negative controls of RA-treated and control hiPSC-CM using appropriate isotype antibodies.

Figure S2. Validation of WB antibodies, related to Figure 2B

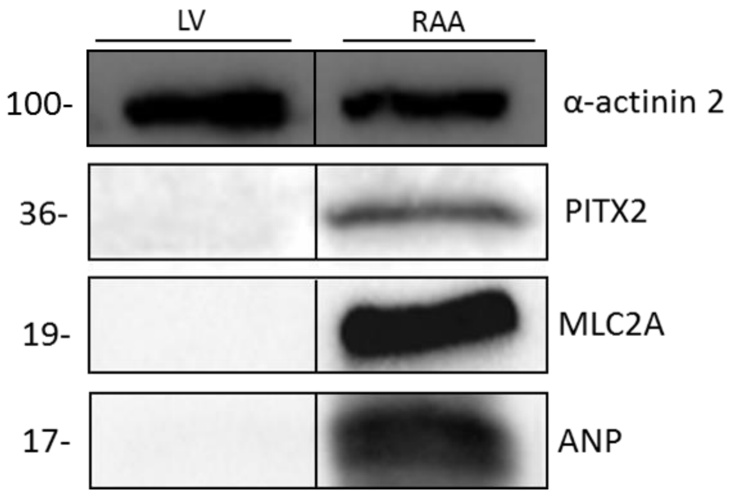

Figure S2. Validation of WB antibodies to test their specificity using human adult cardiac tissue (LV and RAA).

Figure S3. Experiment list for each cell line, related to Figure 2, 4, 5, 6, 7

	FACS	Cell area	qPCR	WB	EHT	IF	AP	TEM	Drug response	CCh: APD_{90} ↓	4-AP: APD_{20} ↑
C25	✓	✓	✓	✓	✓	✓	✓	✓	✓	7.9%±1.6% * n=6	38.3±1.9% ** n=6
ERC18	✓	✓	✓	×	✓	✓	✓	×	✓	20±2.6% * n=5	18.4±3.2% ** n=5
ERC1	✓	×	✓	×	✓	×	✓	×	✓	9.4±1.7% * n=5	21.2±1.8% ** n=5

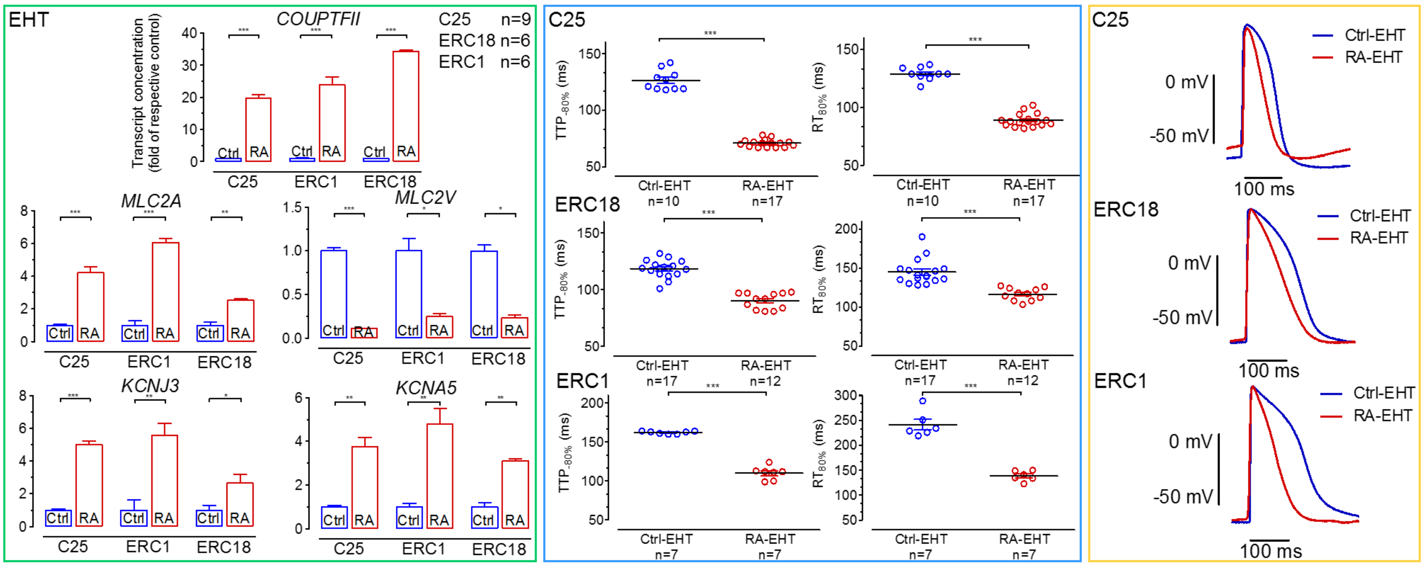

Figure S3. List of experiments performed on each cell line (C25, ERC18 and ERC1). For each cell line, EHT (C25 n=9, ERC1 n=6, ERC18 n=6) expression analysis of atrial specific genes (green box), contractility measurements (Ctrl/RA-EHT: C25 n=10/17, ERC18 n=17/12 and ERC1 n=7/7) (blue box) and AP recordings (yellow box) were performed. In the grey box EHT pharmacological response to atrial selective drug of each cell line (C25 n=6, ERC18 n=5, ERC1 n=5).
n values describe number of EHT obtained from 3 batches of C25 and 2 batches of ERC18 and ERC1. Error bars show mean±SEM.

Figure S4. COUPTFII staining, related to Figure 2 and 3

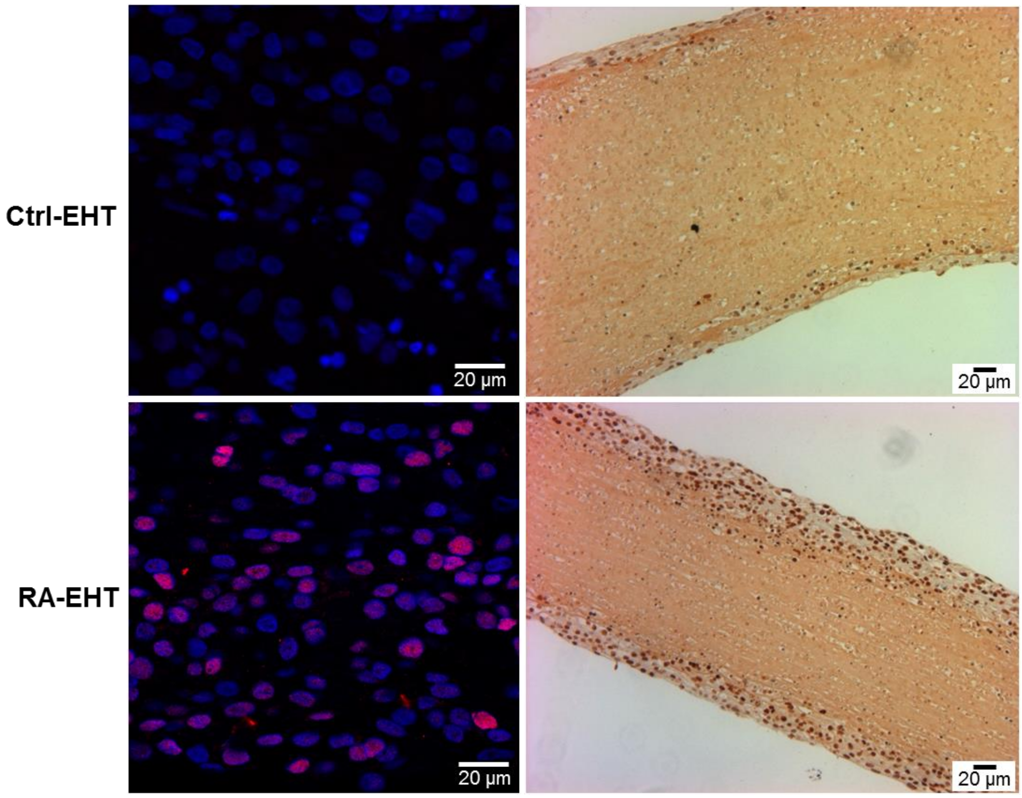

Figure S4. Immunofluorescence labeling (left) and immunohistochemistry (right) of COUPTFII expression in paraffin section of Ctrl- and RA-EHTs.

Figure S5. Live images of Ctrl- and RA-EHTs, related to Figure 3 and 4

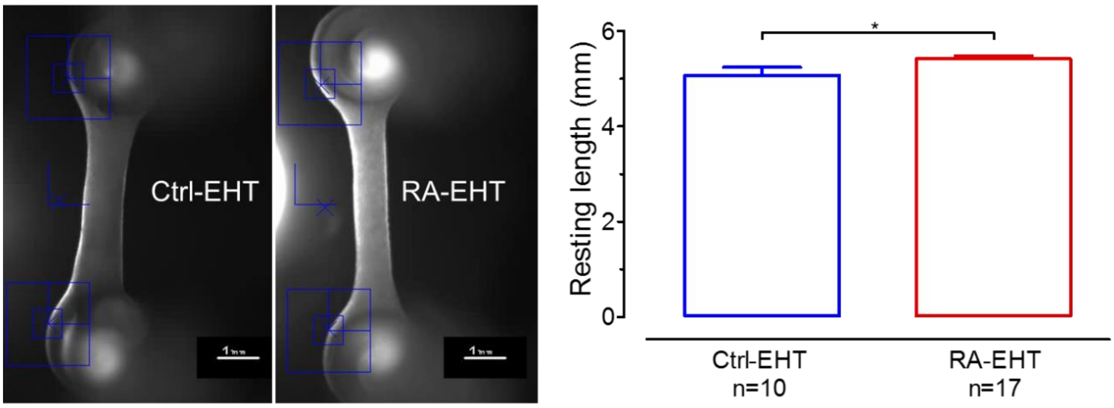

Figure S5. Live images of 20 day-old Ctrl- and RA-EHTs. Scale bar 1 mm. (B, right) RA-EHTs showed slightly higher resting length than Ctrl-EHTs (5.4±0.07 mm vs 5±0.16 mm, n=17/10 from 3 batches; $p<0.05$, unpaired t-test). Error bars show mean±SEM.

Figure S6. Cardiomyocyte organization in EHTs, related to Figure 3 and 4

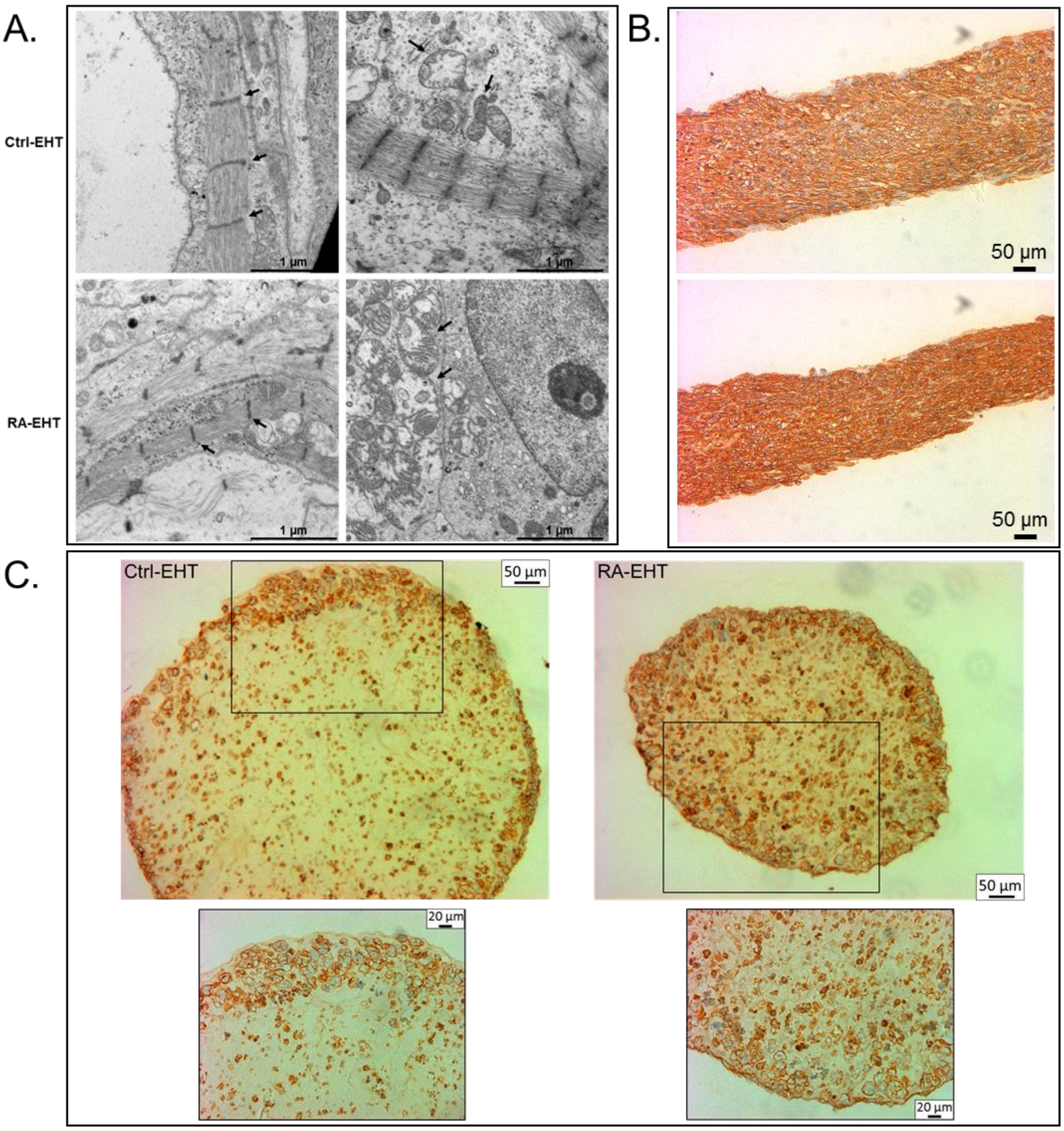

Figure S6. (A) Transmission electron microscopy of 20 day-old EHTs. The cytoplasm of both the Ctrl- and RA-EHTs contained well-developed sarcomeres. Qualitatively, RA-EHTs showed less organized sarcomeres than Ctrl-EHTs. The same finding was shown for fetal human atrial and ventricular cardiac muscle cells (Claycomb et al., 1989). Arrows indicate Z-discs and mitochondria. (B) Immunohistochemistry of α-actinin in paraffin sections of Ctrl- and RA-EHTs. (C) Immunohistochemistry of dystrophin in cross sections of Ctrl- and RA-EHTs.

Figure S7. Functional differences between Ctrl- and RA-EHTs, related to Figure 4, 5, 6 and 7

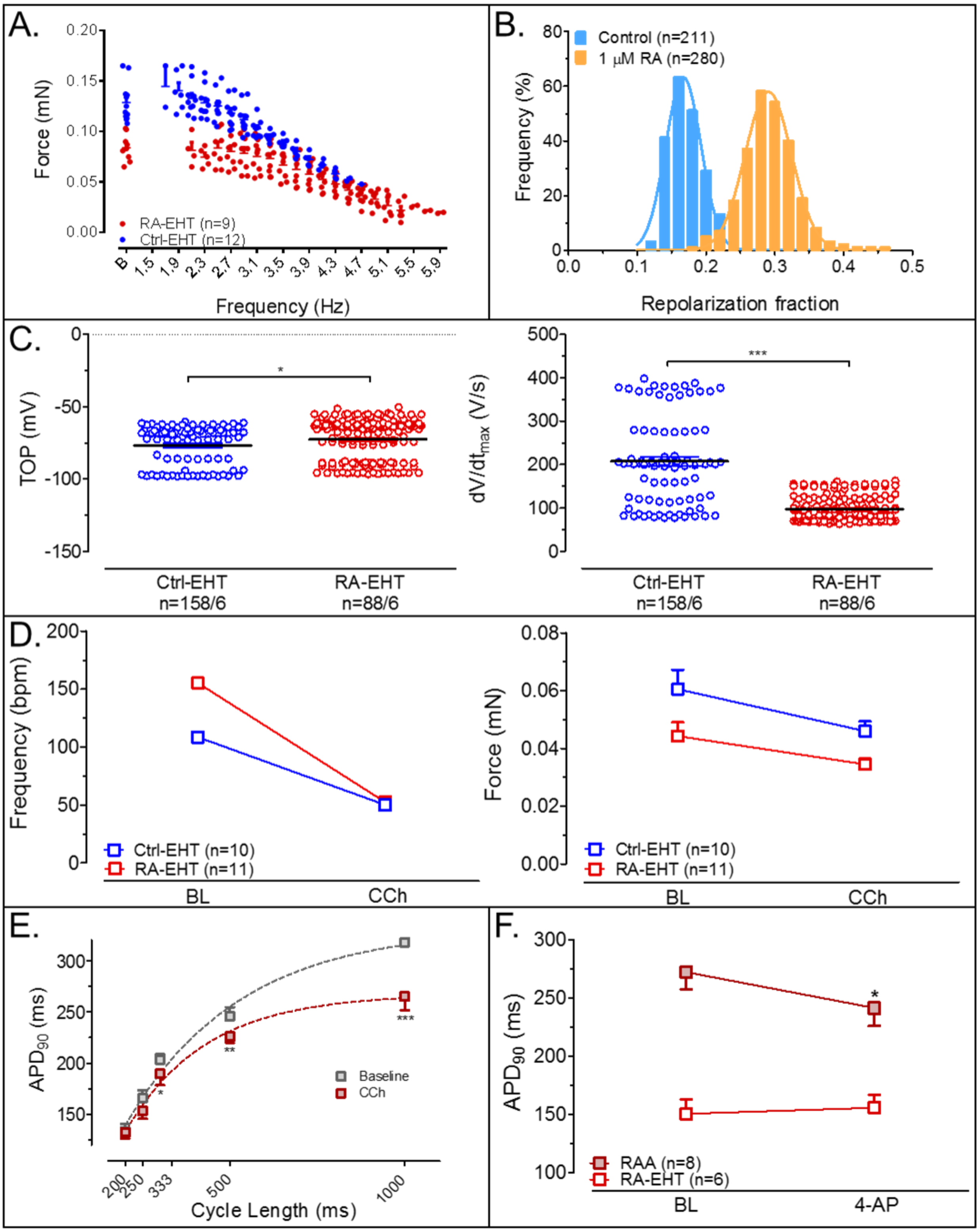

Figure S7. (A) Force-frequency relationship of RA-EHTs (n=9 from 2 batches) and Ctrl-EHTs (n=12 from 2 batches). (B) Frequency distributions for repolarization fraction of Ctrl (n=211 wells, 2 batches) and RA-treated cells (n=280 wells, 2 batches). (C) Take-off potential (left) and upstroke velocity (right) of RA-EHTs (n=88/6, number of impalements/EHTs, 3 batches) and Ctrl-EHTs (n=158/6, number of impalements/EHTs, 3 batches). RA-EHTs showed a less negative take-off potentials (-70±1.1 mV vs. -76±1.5 mV) with slower upstroke velocity (97.6±2.4 V/s vs. 207.6±10.6 V/s). (D) Beating frequency (left) and contractile force (right) of unpaced Ctrl- (n=10 from 3 batches) and RA-EHTs (n=11 from 3 batches) before (baseline, BL) and after CCh (10 µmol/L) exposure. (E) Rate-dependency of CCh-induced action potential shortening in adult human atrium (n=6 patients, $p<0.05$, paired t-test). An exponential function was fitted to the data points. (F) Effect of 4-AP (50 µmol/L) on APD_{90} in RAA (n=8 patients) and RA-EHTs (n=6 from 3 batches).
Error bars show mean±SEM.

2. Supplemental Tables

Table S1. List of RT-qPCR primers, related to Figure 2

Primer Name [HGNC gene name]		Primer Sequence [5'…3'] F: forward, R: reverse	Position CHR (GRCh37/hg19)	Ampl.-length [bp]	Primer T_m [°C]
COUPTFII	Chicken ovalbumin upstream promoter II	F: CCGAGTACAGCTGCCTCAA<br>R: TTTTCCTGCAAGCTTTCCAC	Chr15:96332648-96340955	91	59.41<br>57.11
COUPTFI	Chicken ovalbumin upstream promoter I	F: AAGCCATCGTGCTGTTCAC<br>R: GCTCCTCAGGTACTCCTCCA	Chr5:93586350-93595364	107	58.75<br>60.03
PITX2	Paired-like homeodomain transcription factor 2	F: AGCCATTCTTGCATAGCTCG<br>R: GTGTGGACCAACCTTACGGA	Chr4:110615342-110640316	109	58.41<br>59.61
IRX4	Iroquois Homeobox 4	F: TTGAGAGGTTAAGTTGGGGTTTG<br>R: CCACCCAATTTCTAAAACTAATTACC	Chr5:1878325-1878605	281	58.79<br>56.78
MLC2V	Myosin regulatory light chain 2, ventricular isoform	F: GATGTTCGCCGCCTTCCCCGC<br>R: GCAGCGAGCCCCCTCCTAGT	Chr12:110909841-110921578	106	68.75<br>66.01
MLC2A	Myosin regulatory light chain 2, atrial isoform	F: CACCGTCTTCCTCACACTCTT<br>R: AGGCACTCAGGATGGCTTC	Chr7:44138614-44141608	71	59.66<br>59.39
ANP	Atrial natriuretic peptide	F: ACAGGATTGGAGCCCAGAG<br>R: GGAGCCTCTTGCAGTCTGTC	Chr1:11845501-11847991	109	58.69<br>60.39
SLN	Sarcolipin	F: CTTGGTGTGCCCTCAGAAAT<br>R: TCAGTCAATCCCAGGACCAT	Chr11:107706906-107712530	179	58.08<br>58.03
KCNJ3	Potassium inwardly-rectifying channel, subfamily J, member 3	F: AAAAACGATGACCCCAAAGA<br>R: TGTCGTCATCCTAGAAGGCA	Chr2:154682604-154874329	98	55.46<br>58.15
KCNA5	Potassium voltage-gated channel subfamily A member 5	F: CGAGGATGAGGGCTTCATTA<br>R: CTGAACTCAGGCAGGGTCTC	Chr12:5043632-5047075	186	56.79<br>59.75
SK2	Small conductance calcium-activated potassium channel 2	F: ATGAGCAGCTGCAGGTACAA<br>R: CTAGCTACTCTCTGATGAGG	Chr5:114011956-114540550	1740	59.67<br>53.52
SK3	Small conductance calcium-activated potassium channel 3	F: GTTCTTTCACCCCCTCTTCTTTC<br>R: TTGGCTTGCTTCGGTTCTCT	Chr1:154680180-154887560	123	59.18<br>59.89
GUSB	β-Glucuronidase	F: AAACGATTGCAGGGTTTCAC<br>R: CTCTCGTCGGTGACTGTTCA	Chr2:140043116-142302796	171	57.21<br>59.41

Table S1. Primer sequences used for RT-qPCR assays.

Table S2. Clinical information of patients, related to Figure 5, 6 and 7

	RAA	LV
N	38	30
Gender [m/f]	20 / 18	12 / 18
Age [years]	70.2 ± 2.3	59.4 ± 3.1
BMI [kg/m^2]	26.3 ± 0.8	25.3 ± 0.7
Sinus rhythm	38	30
Hypertension, n	38	16
Diabetes mellitus, n	5	5
Hyperlipidaemia, n	12	7
Coronary artery disease, n	21	3
Valve disease, n	17	20
Hypertrophic obstructive cardiomyopathy, n	0	5
LVEF [%]	59.3 ± 3.3	52.4 ± 3.4
Cardiovascular medication (n)		
Digitalis	2	1
ACE-Inhibitors	9	16
AT_1-blockers	2	5
β-blockers	12	17
Ca^{2+}-channel-blockers	6	1
Diuretics	8	12
Nitrates, n	0	1
Lipid-lowering drugs	11	8

Table S2. Patient characteristics belonging to the atrial and ventricular tissue used for experiments. Abbreviations: AT, angiotensin receptor; LVEF, left ventricular ejection fraction. Mean±SEM.

3. Supplemental Experimental procedures

3.1 Cell size measurement

As previously published (Prondzynski et al., 2017) for cell size analysis, confocal microscopy Zeiss LSM 800 with Airyscan technology was used. HiPSC-CMs were stained for α-actinin 2 (1:800, Sigma) in 96-well plates and >100 images per sample were recorded. Cell sizes from confocal images were measured by using Fiji software (ImageJ). Quality criteria for hiPSC-CM inclusion were set for single cells with well-formed sarcomeres.

3.2 Flow cytometry

For determination of differentiation efficiency, 2 x 10^5 cells were subjected to flow cytometry analysis. Cells were fixed in cold methanol (−20 °C) for 20 min at 4 °C (Breckwoldt et al., 2017) and permeabilized in permealization buffer containing 5% fetal bovine serum, 0.5% saponin (Sigma) ad 0.05% sodium azide. For intracellular staining, cells were stained with directly labeled antibody anti-cardiac Troponin T-FITC, 1:10 dilution (Miltenyi Biotec) in permeabilization buffer for 30 min at 4 °C. As negative control we used appropriate isotype antibody (Figure S1). Stained cells were analyzed using BD FACSCanto II Flow Cytometer and the BD FACSDiva Software 6.0.

3.3 Gene expression analysis

Total RNA was extracted from hiPSC-CMs, cultured for 14-20 days either in ML or EHT format. Extraction of total RNA was performed with RNeasy Mini Kit (Qiagen) according to manufactures instructions. For EHTs, proteinase K (Thermo Scientific) digestion was performed before extraction. Real-time qPCR experiments were performed on three independent Ctrl- and RA-ML/EHT generations. For assessing gene expression by qPCR, cDNA was synthesized from approximately 200 ng of total RNA. RNA was reverse transcribed into cDNA using high capacity cDNA reverse transcription kit (Applied Biosystems). QPCR was performed using Maxima SYBR Green/ROX (Thermo Scientific) on an ABI Prism instrument (Applied Biosystems). Each reaction was performed in triplicates and non-template reaction (replacing cDNA with water) was used as negative control. The cycling parameters were 50 °C for 2 min followed by 95 °C for 10 min, 15 seconds at 95 °C and 1 minute at 60 °C for 40 cycles. mRNA-specific CT values were normalized with CT values for human *GUSB* (beta glucuronidase, housekeeping gene) and with their respective controls (Ctrl-ML and Ctrl-EHT). Relative differences between RA and Ctrl samples were calculated with ΔΔCt method for relative quantifications. Primer sequences are enclosed in the Table S1. Candidates markers for atrial and ventricular phenotype were chosen based on previous publications (Ellinghaus et al., 2005; Gaborit et al., 2007; Wobus et al., 1995).

3.4 Protein analysis by Western Blot

For analysis of proteins, ML were detached with trypsin (3 min, 37°C), spun down (100 g, 5 min) and subsequently dissolved in 100 µL T-PER Tissue Protein Extraction Reagent (ThermoScientific, 78510) with cOmplete Mini EDTA-free protease inhibitor cocktail (Roche Diagnostics, 04693159001). To 10 µl ML lysate, 1xlaemmli buffer was added, subsequently the mixture was heated for 5 min at 95 °C. Proteins were separated by 12% acrylamide/bisacrylamide (29:1, BioRad, 1610156) gels and thereafter transferred onto nitrocellulose (NC) or polyvinylidene fluoride (PVDF) membranes (0.45 µm). Washing steps were performed with TBS-Tween 0.1%, blocking with 5% skim milk powder. Primary antibodies were incubated ON at 4°C in TBS-Tween 0.1%, secondary antibodies for 1 hour at RT in 5% skim milk powder/TBS-Tween 0.1%. Following primary antibodies were used: ANP (NC; 1:10000; Abcam, ab91250), MLC2A (NC; 1:1000; Synaptic Systems, 311011), PITX-2 (NC; 1:250; Invitrogen, PA5-11479), COUPTFII (PVDF; 1:5000; Perseus Proteomics, PP-H7147-00). Following secondary antibodies were used: α-rabbit IgG peroxidase-conjugate (1:10 000; Sigma, A9044), α-mouse IgG peroxidase-conjugate (1:10 000; Sigma, A3682). Visualization was performed with the Clarity Western ECL Substrate (BioRad) at the ChemiDoc imaging system (BioRad).

3.5 Immunohistochemistry and immunofluorescence

14-20 day-old EHTs were fixed in formaldehyde (Roti®-Histofix 4%, Carl Roth, P087.3) ON at 4 °C. After embedding in paraffin, 4 µm thick longitudinal sections were processed for immunohistochemical staining (monoclonal mouse anti-α-actinin 1:800, Sigma A7811 monoclonal rabbit anti-MLC2v 1:200, ProteintecTM, 10906-1-AP; monoclonal mouse anti-MLC2A 1:200, Synaptic systems 311011, monoclonal mouse anti-COUPTFII 1:200; Perseus Proteomics, PP-H7147-00; monoclonal mouse anti-dystrophin 1:200, Millipore MAB1645). All microscopic images were taken on an Axioskop 2 microscope (Zeiss).

For whole mount immunofluorescence staining, fixed EHTs were blocked (6 h in TBS 0.05 M pH 7.4, 10% FCS, 1% BSA, 0.5% Triton X-100), incubated in antibody solution (TBS 0.05 M pH 7.4, 1% BSA, 0.5% Triton X-100) with primary antibodies ON (monoclonal rabbit anti-MLC2v 1:200, ProteintecTM, 10906-1-AP; monoclonal mouse anti-MLC2A 1:200, Synaptic systems 311011; monoclonal mouse anti-COUPTFII 1:200; Perseus Proteomics, PP-H7147-00), washed repeatedly with PBS, incubated in antibody solution with secondary antibodies and other stainings for two hours at RT (Alexa Fluor® 488 goat-anti-rabbit 1:800, Invitrogen; Alexa Fluor® 546 goat-anti-mouse 1:800; DAPI Sigma Aldrich D9564 1:1000), rinsed 2-3 times in PBS and embedded in Fluoromount-G® (SouthernBiotech, 0100-01) in microscope slides (Carl Roth, H884.1). MLs were cultivated for 14 days and then fixed for 20 min at 4 °C and stained accordingly with the exception of using a different permeabilization buffer (1x PBS, milk powder 3% (w/v), Triton X-100). Immunofluorescence images were acquired using Zeiss LSM 800.

3.6 Drugs

CCh and 4-AP were obtained from Sigma-Aldrich (St. Louis, MO, USA). CCh, a muscarinic receptor agonist, was prepared as a 10 mmol/L stock solution in DMSO and stored at -20 ºC. 4-AP was prepared as a 1 mmol/L stock solution in Tyrode's solution, pH adjusted to 7.4 and stored at 4 ºC. All stock solutions were diluted appropriately before use.

3.7 Transmission electron microscopy

For transmission electron microscopy (TEM), EHTs were washed twice in PBS and incubated in 2-butandionemonoxime (Sigma, B0753; 30 mM in PBS, 10 min, 37 °C) to relax sarcomeres and fixed overnight in glutaraldehyde (0.36%, pH 7.0-7.5, 4 °C). Fixed EHTs were removed from silicone racks and subjected to postfixation in osmium tetroxide solution (1%, 2 h; Science Services, 19110), dehydration and embedding in a glycidether-based resin. Ultra-thin sections (50 nm) were prepared and analyzed on a Zeiss LEO 912AB.

3.8 Force-frequency relationship

Force-frequency relationship was measured on 14 day-old EHTs in Gibco DMEM, High Glucose (LS11965092) supplemented with 25 mmol/L HEPES and 1.8 mmol/L $CaCl_2$ pre-equilibrated overnight (37 °C, 7% CO_2, 40% O_2). Following a stabilization period of 30 min in the new medium, contractile force of EHTs under electrical pacing was measured. Spontaneous contraction activity was recorded to determine the starting frequency of the force-frequency relationship. The electrical pacing was performed using a biphasic stimulation characterized by 2.5 V and 4 ms duration. EHT contraction force was recorded for 10 seconds at each frequency. Pacing frequency was varied from 1.5 to 6 Hz in 0.2 Hz steps.

3.9. Flow volumetry analysis

As recently published (Mosqueira et al., 2018) flow volumetry analysis was performed to estimate the volume of hiPSC-CMs after differentiation and after 14 days of EHT culture. In order to establish a calibration curve relating forward light scattered and cell size, calibration beads of known dimensions (2-14.3µm diameter, Spherotech #PPS-6K) were analysed using BD FACSCanto II Flow Cytometer, after excluding debris and duplets/triplets. Only the volume of cardiac troponin T positive cells was measured. Therefore, cell size of hiPSC-CMs was estimated as the volume of a perfect sphere ($V = \frac{\pi}{6} * d^3$) using the calibration curve.

4 Experimental protocol of human samples

Human tissue samples were transported in Ca^{2+}-free transport solution at 20–25 °C for maximum 30 min, composition in mmol/L: 100.0 NaCl, 10.0 KCl, 1.2 KH_2PO_4, 5.0 $MgSO_4$, 50 taurin, 5 MOPS, 30 butanedione monoxime (BDM), pH 7.0. Before the start of each measurement, tissue samples were superfused with Tyrode's solution at 36.5±0.5 °C for at least 30 minutes.

4. Supplemental References

Breckwoldt, K., Letuffe-Brenière, D., Mannhardt, I., Schulze, T., Ulmer, B., Werner, T., Benzin, A., Klampe, B., Reinsch, M.C., Laufer, S., et al. (2017). Differentiation of cardiomyocytes and generation of human engineered heart tissue. Nat. Protoc. *12*, 1177–1197.

Claycomb, W.C., Delcarpio, J.B., Guice, S.E., and Moses, R.L. (1989). Culture and characterization of fetal human atrial and ventricular cardiac muscle cells. Vitr. Cell. Dev. Biol. Tissue Cult. Assoc. *25*, 1114–1120.

Ellinghaus, P., Scheubel, R.J., Dobrev, D., Ravens, U., Holtz, J., Huetter, J., Nielsch, U., and Morawietz, H. (2005). Comparing the global mRNA expression profile of human atrial and ventricular myocardium with high-density oligonucleotide arrays. J. Thorac. Cardiovasc. Surg. *129*, 1383–1390.

Gaborit, N., Le Bouter, S., Szuts, V., Varro, A., Escande, D., Nattel, S., and Demolombe, S. (2007). Regional and tissue specific transcript signatures of ion channel genes in the non-diseased human heart. J. Physiol. *582.2*, 675–693.

Mosqueira, D., Mannhardt, I., Bhagwan, J.R., Lis-Slimak, K., Katili, P., Scott, E., Hassan, M., Prondzynski, M., Harmer, S.C., Tinker, A., et al. (2018). CRISPR/Cas9 editing in human pluripotent stem cell-cardiomyocytes highlights arrhythmias, hypocontractility, and energy depletion as potential therapeutic targets for hypertrophic cardiomyopathy. Eur. Heart J. *0*, 1–16.

Prondzynski, M., Krämer, E., Laufer, S.D., Shibamiya, A., Pless, O., Flenner, F., Müller, O.J., Münch, J., Redwood, C., Hansen, A., et al. (2017). Evaluation of MYBPC3 trans-Splicing and Gene Replacement as Therapeutic Options in Human iPSC-Derived Cardiomyocytes. Mol. Ther. - Nucleic Acids *7*, 475–486.

Wobus, A.M., Rohwedel, J., Maltsev, V., and Hescheler, J. (1995). Development of Cardiomyocytes Expressing Cardiac-Specific Genes, Action Potentials, and Ionic Channels during Embryonic Stem Cell-Derived Cardiogenesis. Ann. N. Y. Acad. Sci. *752*, 460–469.
